# Exploratory analysis of nasal and gut microbial community structure in preschool children with recurrent wheeze

**DOI:** 10.1007/s00248-026-02867-3

**Published:** 2026-09-01

**Authors:** Silvia Gschwendtner, Nicole Maison, Sabina Illi, Erika von Mutius, Ilona Rosenboom, Burkhard Tümmler, Anna-Maria Dittrich, Markus Weckmann, Mustafa Abdo, Benjamin Waschki, Matthias V. Kopp, Gesine Hansen, Folke Brinkmann, Klaus Rabe, Bianca Schaub, Michael Schloter, the ALLIANCE Study Group, Mira Bürk, Sybille Contento, Markus Ege, Alexander Hose, Constanze Jakwerth, Lena Lagally, Jimmy Omony, Carsten Schmidt-Weber, Lena Ullemeyer, Esther Zeitlmann, Thomas Bahmer, Heike Biller, Constantin Blanke-Roeser, Karoline I. Gaede, Wiebke Hagedorn, Nikolas Jacobs, Anne-Marie Kirsten, Inke R. König, Lea Kronziel, Gyde Nissen, Wasifa Nurieva, Frauke Pedersen, Loana Penner, Isabell Ricklefs, Silke Szymczak, Vera Veith, Henrik Watz, Christopher Wirks, Marie Bickes, Mifflin-Rae Calvero, David S. DeLuca, Svenja Gaedcke, Ruth Grychtol, Anika Habener, Christine Happle, Adan Chari Jirmo, Bin Liu, Lennart Riemann, Svenja Foth, Inga Jerrentrup, Harald Renz, Chrysanthi Skevaki, Stefanie Weber, Miguel AAlejandre Alcazar, Lena Keufken, Silke van Koningsbruggen-Rietschel, Ernst Rietschel, Tobias Trojan, Jan-Christoph Thomassen

**Affiliations:** 1 Research Unit of Comparative Microbiome Analysis, Helmholtz Munich, German Research Center for Environmental Health, Neuherberg, Germany; 2 Department of Pediatric Allergology, Dr. von Hauner Children’s Hospital, Ludwig Maximilians University, Munich, Germany; 3 Comprehensive Pneumology Center Munich (CPC-M), German Center for Lung Research (DZL), Munich, Germany; 4 Institut für Asthma- und Allergieprävention (IAP), Helmholtz Munich, German Research Center for Environmental Health, Neuherberg, Germany; 5 Department of Pediatric Pneumology, Allergology and Neonatology, Hannover Medical School, Hanover, Germany; 6 Biomedical Research in Endstage and Obstructive Lung Disease Hannover (BREATH), German Center for Lung Research (DZL), Hanover, Germany; 7 Cluster of Excellence RESIST (EXC 2155), German Research Foundation (DFG), Hannover Medical School, Hannover, Germany; 8 Airway Research Center North (ARCN), German Center for Lung Research (DZL), Borstel, Lübeck and Großhansdorf, Germany; 9 Department of Paediatric Pneumology & Allergology, University Clinics Schleswig-Holstein (UKSH), Lübeck, Germany; 10 Division of Epigenetics in Chronic Lung Disease, Priority Area Chronic Lung Diseases, Leibniz Lung Center, Research Center Borstel, Borstel, Germany; 11 LungenClinic Grosshansdorf GmbH, Grosshansdorf, Germany; 12 Department of Pediatric Respiratory Medicine, Inselspital, University Children’s Hospital of Bern, University of Bern, Bern, Switzerland; 13 German Center for Child and Adolescent Health (DZKJ), Dr. von Hauner Children’s Hospital, LMU, Munich, Germany; 14 Chair for Environmental Microbiology, Technical University of Munich, Freising, Germany

**Keywords:** Gut-airway axis, Nasal microbiome, Gut microbiome, Recurrent preschool wheeze, Exploratory pilot study, ALLIANCE cohort

## Abstract

Early-life wheezing has been associated with microbial alterations along the gut-airway axis, yet few studies have examined bacterial communities in both compartments within the same children. This cross-sectional exploratory pilot study (n=25) characterized nasal and stool bacterial communities in children aged 1–4 years with recurrent wheeze and healthy controls using 16S rRNA gene metabarcoding. Across participants, nasal and stool bacteriomes were highly individualized and taxonomically diverse. Overall richness, evenness, and global community composition did not differ significantly between healthy controls and children with recurrent wheeze in either compartment. Nasal communities in the recurrent-wheeze group showed greater within-group dispersion, although the comparison did not reach the conventional significance threshold (p=0.054). Exploratory distance-based stratification identified a subgroup with nasal profiles farther from the healthy-control centroid; these profiles had lower richness and evenness, higher *Moraxella* abundance, and lower abundances of *Prevotella* spp., *Alloprevotella*, and *Veillonella*. No statistically supported genus-level differences were detected in stool samples. Community-assembly and co-occurrence analyses yielded group-specific descriptive patterns in both compartments. Different sets of cross-compartment correlations passed the multiple-testing threshold in healthy controls and children with recurrent wheeze, including correlations of stool *Lactococcus* with several nasal taxa in the recurrent-wheeze group. Because these analyses were performed in small groups, they are considered hypothesis-generating. These findings identify nasal microbial heterogeneity and exploratory differences in community organization in preschool children with recurrent wheeze, while providing no evidence for causal or *Moraxella*-driven effects. Larger longitudinal studies with functional measurements are required for validation.

**Supplementary Information:**

The online version contains supplementary material available at https://doi.org/10.1007/s00248-026-02867-3.

## Introduction

Human microbiota play a central role in regulating local and systemic immune function. A prominent example of such systemic crosstalk is the axis between the gut and the respiratory system, describing bidirectional communication between intestinal and airway microbiomes mediated by microbial metabolites and immune modulation [1–3]. Increasing evidence indicates that this axis is associated with asthma pathogenesis, particularly during early life when immune development is highly sensitive to environmental influences [4, 5].

The gut microbiome begins to establish at birth and undergoes rapid succession during infancy. In human studies, stool samples commonly serve as a non-invasive proxy for intestinal microbial communities. Early colonization is influenced by mode of delivery, diet, and environmental exposures [6]. During early infancy, *Bifidobacterium* species dominate, particularly in breastfed infants [7, 8]. With the introduction of complementary feeding, microbial diversity increases with greater representation of *Bacteroides* and Firmicutes including *Blautia*, *Faecalibacterium*, and *Roseburia* [7]. By approximately three years of age, the microbiome stabilizes toward an adult-like configuration dominated by *Bacteroides, Prevotella,* and various Firmicutes [8, 9].

The respiratory microbiome develops along a dynamic trajectory. Nasal swabs characterize the nasal upper-airway niche and may provide information on microbial colonization at the entrance of the respiratory tract. They do not, however, directly measure lower-airway communities. In early infancy, nasal communities are typically dominated by *Staphylococcus* and *Corynebacterium* [10], while *Alloiococcus* and *Moraxella* increase during the first year of life [11]. Early colonization with *Streptococcus* and *Haemophilus* has been associated with viral respiratory tract infections, although these genera may also occur during normal microbial succession [11]. With increasing age, nasal communities diversify further, with taxa such as *Dolosigranulum* and *Corynebacterium* becoming more prominent by two to three years of age [12]. Early upper-airway microbial composition and diversity have been linked to immune development and allergic sensitization [13].

Consistent with these observations, early microbial dysbiosis in either the intestinal or airway microbiome has been associated with wheezing illnesses and subsequent asthma development [11, 14–18]. While these findings support the concept of an axis of the gut and the respiratory system, most studies have examined the gut or respiratory microbiome separately, and investigations assessing microbial communities of both organs in parallel remain rare.

To address this gap, we characterized stool and nasal bacterial communities in children aged 1–4 years with recurrent wheeze (n=17) and healthy controls (n=8) in a cross-sectional exploratory pilot study. Stool samples and nasal swabs served as proxies for intestinal and nasal upper-airway microbiomes, respectively. Using 16S rRNA gene metabarcoding, we assessed community composition, diversity, assembly patterns, co-occurrence networks, and cross-compartment correlations. Given the limited sample size and cross-sectional design, these analyses were intended to generate hypotheses rather than establish causal microbial signatures of wheeze or asthma risk.

## Materials and Methods

### Study Design

The ALLIANCE cohort of the German Center for Lung Research (DZL) is a prospective multicenter asthma cohort recruiting in five pediatric specialist centers (Hannover, Luebeck, Munich, Marburg and Cologne) and two adult specialist centers (LungenClinic Grosshansdorf and Research Centre Borstel), registered at ClinicalTrials.gov (pediatric arm NCT02496468, adult arm NCT02419274) and ethically approved across all study centers. Parents of study participants aged < 18 years and study participants aged ≥ 18 years gave written informed consent.

Eligibility for this exploratory pilot study required age 1–4 years, availability of paired nasal swab and stool samples, and wheeze status. The recurrent-wheeze group (hereafter termed “wheezers” for brevity) comprised 19 children selected from 221 children with at least two wheezing episodes during the preceding 12 months, based on parental reports. Two children were excluded because their nasal swabs yielded no bacterial sequencing reads; their corresponding stool samples were also excluded to maintain paired analyses, resulting in 17 wheezers. Eight age- and sex-matched healthy controls (HC) were selected from 54 controls who had never been diagnosed with asthma or preschool wheeze, irrespective of other allergic diseases. This definition does not exclude every possible isolated episode of acute wheeze. Patient characteristics are summarized in Table 1. Details regarding study design, parameter definitions, inclusion criteria, and methods are provided in the Supplementary Material and have been published previously [19].

**Table 1:** Summary of baseline characteristics of the study cohort

Characteristic	Overall	Healthy	Wheeze	*p* value ^1^
	*N* = 25	*N* = 8	*N* = 17	
Age (year)	2.70 (1.90–3.30)	2.65 (1.90–2.85)	3.00 (1.90–3.40)	0.209
Female	8 (32%)	4 (50%)	4 (24%)	0.359
BMI (kg/m^2^)	16.80 (15.50–17.94)	16.50 (15.35–17.30)	16.98 (15.50–18.20)	0.268
Any sibling	16 (64%)	4 (50%)	12 (71%)	0.394
Daycare	23 (92%)	8 (100%)	15 (88%)	> 0.999
Pet in household (cat/dog)	9 (36%)	2 (25%)	7 (41%)	0.661
Asthma or recurrent spastic/obstructive bronchitis - DD	2 (8.3%)	0 (0%)	2 (13%)	0.536
Missing	1	0	1	
Hayfever - DD	1 (4.2%)	0 (0%)	1 (5.9%)	> 0.999
Missing	1	1	0	
Eczema - DD	2 (8.3%)	0 (0%)	2 (13%)	0.536
Missing	1	0	1	
Food allergy - DD	0 (0%)	0 (0%)	0 (0%)	-- ^2^
Missing	1	1	0	
Birthmode				0.095
Vaginal delivery	15 (60%)	7 (88%)	8 (47%)	
Primary cesarean section	6 (24%)	0 (0%)	6 (35%)	
Secondary cesarean section	2 (8.0%)	1 (13%)	1 (5.9%)	
Operative vaginal delivery	2 (8.0%)	0 (0%)	2 (12%)	
Any exclusive breastfeeding ever	19 (76%)	5 (63%)	14 (82%)	0.344
Any hypoallergenic formula ever	9 (36%)	1 (13%)	8 (47%)	0.182
Prior RSV infection ever	7 (32%)	0 (0%)	7 (47%)	0.051
Missing	3	1	2	
Antibiotics given for recurrent cough ever	1 (33%)	NA	1 (33%)	-- ^2^
Missing	22	8	14	
Systemic steroids ever	9 (53%)	NA	9 (53%)	-- ^2^
Missing	8	8	0	
ICS ever	13 (76%)	NA	13 (76%)	-- ^2^
Missing	8	8	0	
Hb (g/dl)	11.60 (11.40–12.30)	11.60 (11.30–12.30)	11.90 (11.45–12.25)	0.365
Missing	2	1	1	
Thrombocytes (10^3^/μl)	374 (328–443)	328 (263–367)	403 (372–443)	0.045
Missing	2	1	1	
Leucocytes (10^3^/μl)	9.44 (8.23–11.70)	10.00 (5.24–14.00)	9.27 (8.24–11.65)	0.841
Missing	2	1	1	
Neutrophils (%)	40 (33–55)	38 (31–58)	42 (36–55)	0.947
Missing	2	1	1	
Lymphocytes (%)	42 (34–54)	53 (27–60)	40 (36–47)	0.593
Missing	2	1	1	
Eosinophils (%)	1.95 (1.00–3.00)	2.50 (1.00–3.50)	1.50 (0.78–2.40)	0.243
Missing	3	1	2	
Basophils (%)	0.20 (0.00–0.70)	0.00 (0.00–0.20)	0.55 (0.08–1.00)	0.036
Missing	4	1	3	
IgE (IU/ml)	39.0 (9.1–73.8)	47.5 (24.0–61.4)	19.2 (9.0–82.0)	0.583
Missing	2	0	2	
Atopy (spec. IgE to any allergen ≥ 0.7 kU/L)	8 (32%)	3 (38%)	5 (29%)	> 0.999

Values are summarized as median and interquartile range (Q1–Q3) in brackets, or as total numbers per condition level and percentage in brackets

*DD* parental report of doctor’s diagnosis

^1^Kruskal–Wallis rank sum test; Fisher's exact test

^2^p value not calculated due to insufficient variability (single-level data) and/or missing values

### Microbiome Analysis

Details on microbiome sample processing and subsequent data analysis are provided in Supplementary Material. Briefly, DNA was extracted via ultrasonication (nose) and a phenol-chloroform-based protocol (stool). Amplicon sequencing of the V4 hypervariable region of the 16S rRNA gene was performed on a MiSeq Illumina instrument using the universal eubacterial primers 515F/806R [20, 21]. To identify potential contaminants deriving from DNA extraction and library preparation, both extraction and PCR no template control samples (each 4) were processed together with biological samples. All biological and control samples were sequenced in a single sequencing run to avoid batch effects related to processing steps. Sequence data generated in this study have been deposited in the NCBI Sequence Read Archive (SRA) under BioProject accession number PRJNA1499410.

Sequence processing was performed using DADA2 v 1.30 [22], followed by taxonomic assignment via SILVA v138.1. Reads classified as mitochondrial or chloroplast in origin, or lacking phylum-level annotation were excluded. All blank controls were processed and sequenced alongside the biological samples. After DADA2 denoising, blank controls yielded 0–6 reads per sample, corresponding to <0.01% of the mean sequencing depth of the biological samples (74,896 reads). Across all blank controls, seven amplicon sequence variants (ASV) were detected and assigned to the genera *Agathobacter*, *Bacteroides*, *Faecalibacterium*, *Moraxella*, *Pediococcus*, and *Streptococcus*, while one ASV remained unclassified at the phylum level. None of these ASV was detected in more than one blank control. Only ASV 653 (*Pediococcus*) was consistently identified as a potential contaminant using prevalence-based decontam analysis at thresholds of 0.1 and 0.5 [23] and was therefore removed from all biological samples. Quality-control information for the blank controls, including DNA concentration, read numbers before and after DADA2 denoising, detected ASV, their occurrence in biological samples, and their mean relative abundance, is provided in Supplementary Table S1. Given the extremely low sequencing depth of the blank controls, the absence of recurrent ASV across blank controls, and the decontam results, there was no evidence of systematic contamination by the remaining ASV, which were therefore retained in the biological-sample dataset. Following removal of ASV 653, the dataset comprised 3,744,773 reads assigned to 2,177 ASVs for subsequent analyses.

All plots and statistics were performed in R version 4.5.1 (https://www.R-project.org). Subsequent bioinformatic analyses were performed to characterize microbial communities in HC and wheezers, including assessments of alpha and beta diversity, core microbiome structure, within-group variability, and microbial biomarkers (ANCOM-BC2). Additional analyses explored associations between microbial taxa, individual variability and host factors, cross-compartment correlations, microbial community assembly processes (βNTI and RCbray framework), and bacterial co-occurrence networks (NetCoMi). Furthermore, wheezers were stratified into healthy-like and divergent microbiome profiles and their associated features were examined. Detailed protocols are provided in Supplementary Material.

## Results

### Cohort Characteristics

The study comprised 25 preschool participants: 17 children with recurrent wheeze and 8 healthy controls (HC) (Table 1). Age, sex, early-life and environmental exposures, and most recorded clinical variables were not statistically different between groups. Prior RSV infection nevertheless showed a clinically relevant imbalance: it was reported in 7 of 15 wheezers with available data and in 0 of 7 controls with available data (Fisher’s exact p=0.051). Antibiotic information was largely missing, and systemic or inhaled steroid exposure was recorded only for wheezers. Wheezers had higher thrombocyte and blood basophil counts (Fisher’s exact p=0.045 and p=0.036, respectively); no statistically supported differences were observed for the other measured hematological or immunological parameters.

### Overall nasal and stool diversity is similar between groups, with greater nasal dispersion in children with recurrent wheeze

**Fig. 1:**
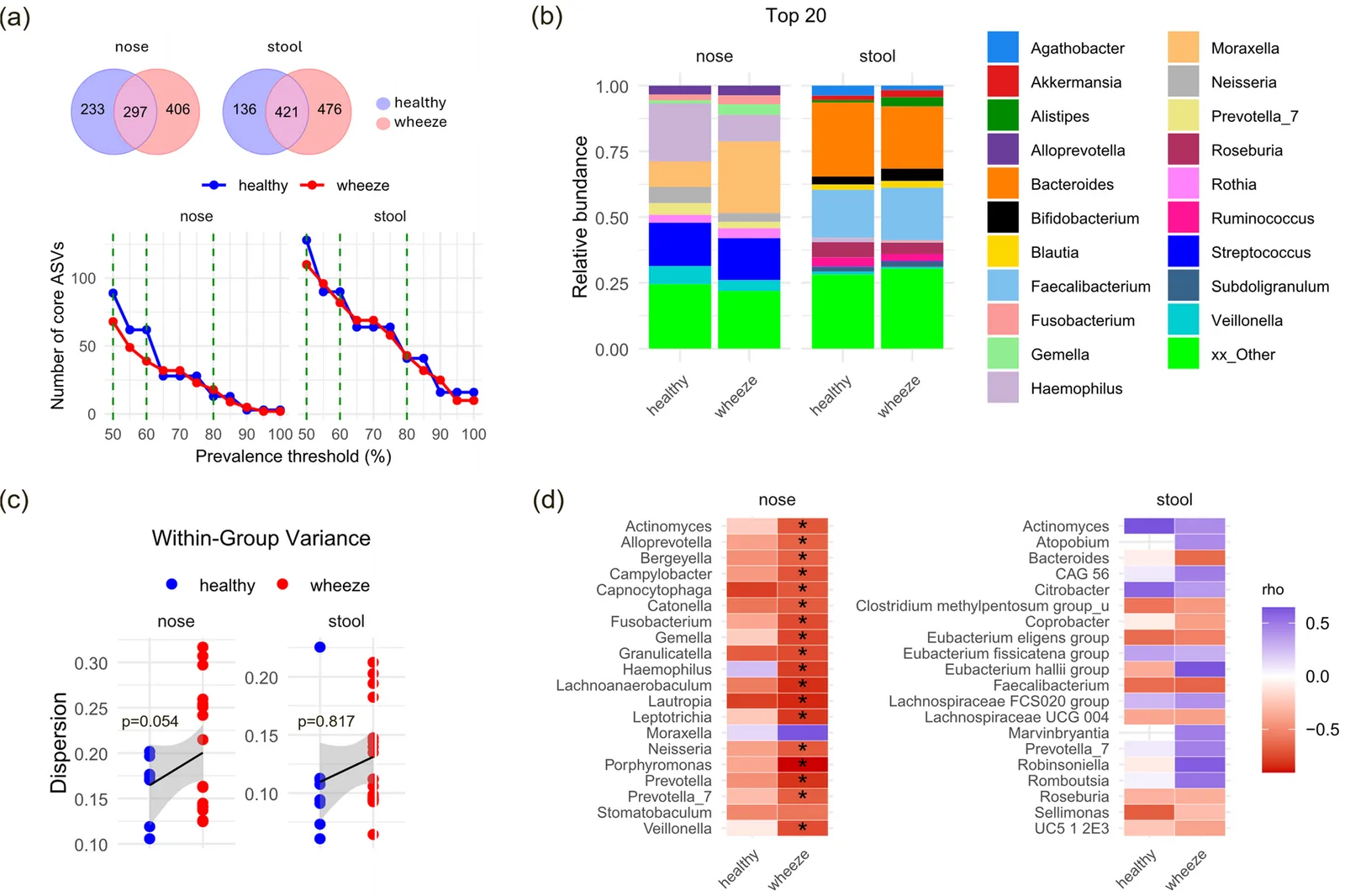
Nasal and gut microbiome composition in healthy (*n* = 8) and wheezing (*n* = 17) children. (**a**) Shared and unique ASV and core ASV across prevalence thresholds. Top: Venn diagrams showing shared and group-specific ASV in nasal and stool samples. Bottom: Number of core ASV across prevalence thresholds (50–100%), defined as ASV present in ≥ X% of samples within each study group. (**b**) Relative abundance of the 20 most abundant genera. No significant differences in genus abundance were detected between groups (ANCOM-BC2; Benjamini–Hochberg-adjusted *p* < 0.05). Effect sizes, 95% CI, and p values are listed in Supplementary Table S3. (**c**) Within-group variation (beta-dispersion) calculated from weighted UniFrac distances. Statistical significance was assessed using Levene’s test and confirmed using generalized linear mixed-effects models with Benjamini–Hochberg adjustment for multiple testing. (**d**) Spearman correlations between beta-dispersion and bacterial genera. Only the 20 strongest correlations, ranked by Spearman’s correlation coefficient (rho), are shown. Asterisks indicate statistically significant correlations (Benjamini–Hochberg-adjusted *p* < 0.05). Correlations were calculated using Spearman’s rank correlation test. Effect sizes, 95% CI, and p values are listed in Supplementary Table S4

Across participants, nasal and stool bacteriomes were taxonomically diverse but highly individualized. The nasal bacteriome comprised 936 ASV and stool samples 1033 ASV, with overlap but more wheezer-specific variants (Fig. 1a). Group-specific core ASV decreased rapidly with increasing prevalence thresholds in both compartments, indicating limited overlap between individuals (Fig. 1a; Supplementary Table S2). Nevertheless, alpha diversity metrics indicated comparable richness and evenness between HC and wheezers in both compartments (Supplementary Fig. S1a), and NMDS analysis of weighted UniFrac distances showed no group separation (Supplementary Fig. S1b).

Nasal bacterial communities were dominated by *Haemophilus*, *Moraxella*, *Streptococcus*, *Veillonella*, and *Prevotella*-related taxa (Fig. 1b). *Moraxella *occasionally dominated individual profiles (Supplementary Fig. S2), but its abundance did not differ significantly between groups (β = 0.57, 95% CI [−0.53, 1.67], adjusted p = 0.308). Among 11 *Moraxella* ASV, one variant closely related to *Moraxella nonliquefaciens* predominated across samples (Supplementary Fig. S3) but occurred more consistently in HC despite its lower relative abundance (Supplementary Table S2). Beta-dispersion was higher in wheezers than in HC, although the comparison was of borderline statistical support (Levene test, F = 3.94, p=0.054; Fig. 1c). Furthermore, wheezers showed significant negative associations between dispersion and nasal genera, including Neisseria (rho = −0.69, 95% CI [−0.94, −0.21], adjusted p = 0.032), Prevotella (rho = −0.82, 95% CI [−0.95, −0.44], adjusted p = 0.003), and Veillonella (rho = −0.74, 95% CI [−0.89, −0.34], adjusted p = 0.015), while Moraxella was positively associated but not statistically significant (rho = 0.64, 95% CI [0.07, 0.92], adjusted p = 0.064).

Stool communities showed higher richness (β = 0.23, 95% CI [0.16, 0.29], adjusted p<0.001) and evenness (β = 0.67, 95% CI [0.21, 1.13], adjusted p=0.019) than nasal communities (Supplementary Fig. S1) and were dominated by *Bacteroides* and *Faecalibacterium* (>43% of reads; Fig. 1b). No genus-level differences between wheezers and HC were statistically supported after correction for multiple testing. Stool within-group dispersion was lower than nasal dispersion (β = −0.69, 95% CI [−0.90, −0.47], adjusted p<0.001; Fig. 1c), and the difference between wheezers and HC was not statistically significant (Levene test, F = 0.021, p=0.817). Associations between stool dispersion and individual genera were likewise not supported after correction for multiple comparisons (Fig. 1d).

Despite substantial inter-individual variability, several host-microbiome associations were observed. In the nose, leukocyte levels correlated negatively with *Corynebacterium *(rho = −0.69, 95% CI [−0.84, −0.34], adjusted p = 0.013),* Dolosigranulum *(rho = −0.58, 95% CI [−0.81, −0.06], adjusted p = 0.048) and* Moraxella *(rho = −0.61, 95% CI [−0.79, −0.22], adjusted p = 0.048) (Supplementary Fig. S4a). In stool, immune parameters were not associated with bacterial diversity. However, age correlated negatively with *Bifidobacterium* (rho = −0.73, 95% CI [−0.91, −0.39], adjusted p = 0.001), while *Ruminococcus* and *Agathobacter* were associated with gender (rho = 0.61, 95% CI [0.30, 0.81], adjusted p = 0.032; and rho = 0.71, 95% CI [0.48, 0.86], adjusted p = 0.003, respectively). Beta-dispersion, used as a measure of individual variability, was not associated with measured host parameters (Supplementary Fig. S4b).

### Exploratory distance-based stratification identifies heterogeneous microbial profiles among wheezers

To explore heterogeneity among wheezers, we performed an explicitly exploratory distance-based stratification. Profiles within the 90% quantile of Euclidean distances around the HC centroid in ordination space were labelled “healthy-like,” whereas profiles beyond this threshold were labelled “community-divergent.” These labels describe relative similarity to the HC profiles; they do not demonstrate naturally occurring or independently validated clusters, and the threshold was not optimized against clinical outcomes. The resulting groups did not differ in the recorded demographic or clinical variables (Supplementary Table S3).

**Fig. 2:**
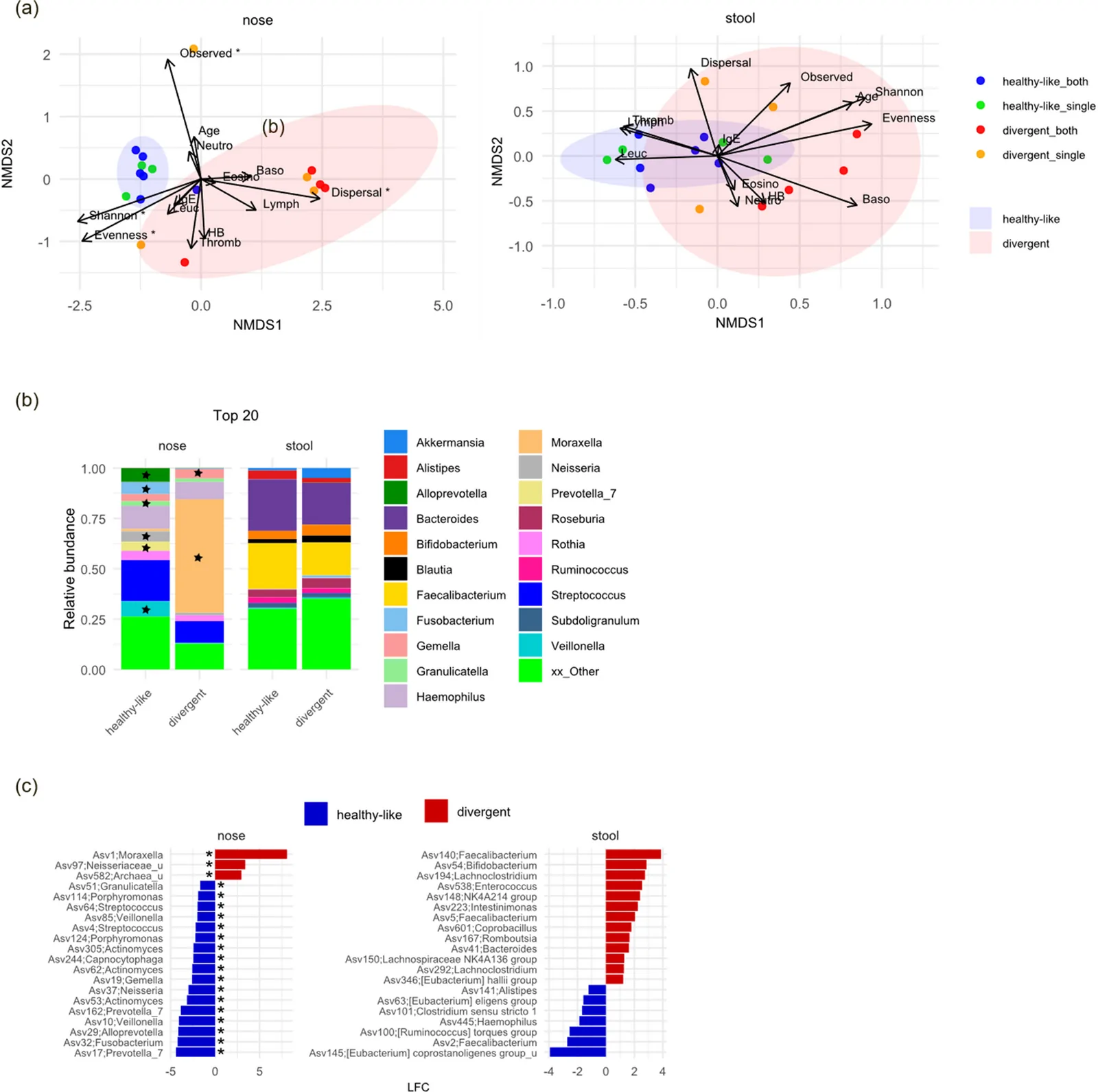
Microbial signatures of exploratory distance-defined wheezer subgroups. (**a**) Non-metric multidimensional scaling (NMDS) ordination based on Bray–Curtis distances, with alpha-diversity metrics (observed ASV, evenness and Shannon index), beta-dispersion and immune variables fitted as environmental vectors. Asterisks indicate vectors significantly associated with community composition (permutation test within the envfit function, *p* < 0.05). Vector length reflects the strength of the association with the ordination, and vector direction indicates the gradient of increasing values across the NMDS space. Blue and red symbols represent subjects classified as healthy-like and community-divergent wheezers, respectively, in both nasal and gut microbiomes; green and orange symbols represent subjects classified as healthy-like and community-divergent wheezers, respectively, in only one compartment (nasal or gut). Effect sizes, 95% CI, and p values of alpha diversity and beta-dispersion analysis are listed in Supplementary Table S6. (**b**) Relative abundance of the 20 most abundant genera. Asterisks indicate significant differences between healthy-like and community-divergent wheezers (ANCOM-BC2; Benjamini–Hochberg-adjusted *p* < 0.05). Effect sizes, 95% CI, and p values are listed in Supplementary Table S7. (**c**) Log-fold changes (LFC) estimated by ANCOM-BC2 for the 20 ASV with the largest effect sizes differentiating the exploratory distance-defined subgroups. Asterisks indicate statistically significant differences after Benjamini–Hochberg adjustment (adjusted p<0.05). Effect sizes, 95% CI, and p values are listed in Supplementary Table S8

In the nasal bacteriome, 9 of 17 wheezers had a profile classified as healthy-like and 8 had a profile classified as community-divergent. The distance-defined divergent subgroup had higher individual variability but lower observed ASV richness, Shannon diversity, and evenness (Fig. 2a; Supplementary Table S4), together with higher *Moraxella* (LFC = 9.04, 95% CI [7.12, 10.95], adjusted p < 0.001) and lower commensals including *Alloprevotella* (LFC = −3.72, 95% CI [−4.77, −2.68], adjusted p < 0.001), *Prevotella*_7 (LFC = −3.99, 95% CI [−4.76, −3.21], adjusted p < 0.001), and *Veillonella* (LFC = −3.15, 95% CI [−4.11, −2.21], adjusted p < 0.001) abundances at genus (Fig. 2b) and ASV level (Fig. 2c). These comparisons describe the exploratory subgroups and do not establish *Moraxella* as a driver of the observed profiles.

In stool bacterial communities, 7 wheezers were classified as community-divergent based on their distance from the HC centroid. Differences between the distance-defined subgroups were less pronounced than in nasal samples, and no genus-level differences were statistically supported after correction for multiple testing (Fig. 2b). Recorded baseline parameters did not differ between stool-based subgroups (Supplementary Table S3).

Partial overlap between compartments was observed: four of the eight nasal-divergent wheezers were also classified as divergent in stool, whereas the remaining individuals showed healthy-like stool profiles. Similarly, six of nine healthy-like wheezers displayed concordant bacterial signatures in both compartments.

### Exploratory community-assembly and co-occurrence patterns in nasal and stool bacteriomes

**Fig. 3:**
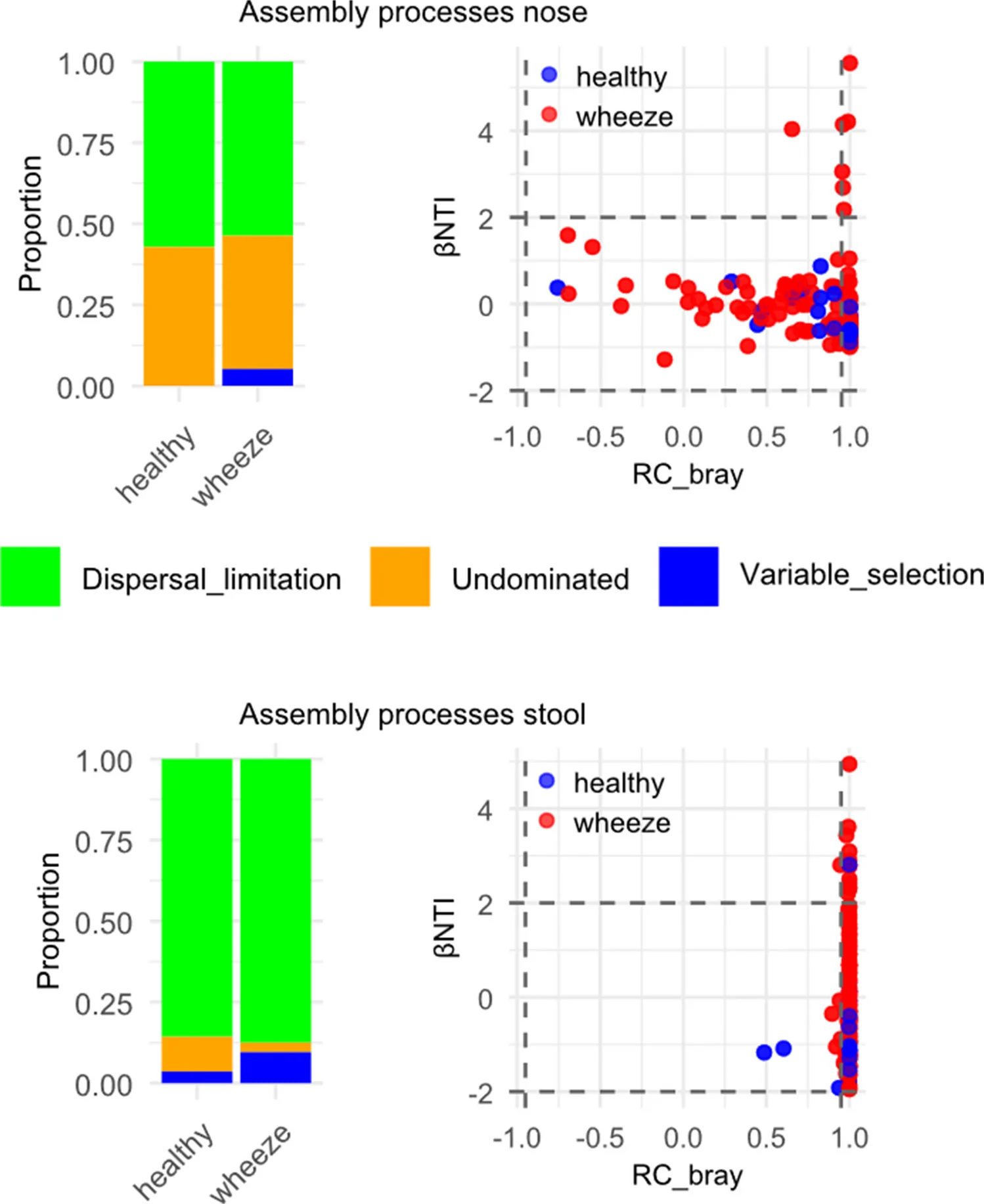
Community assembly processes in the nasal and gut microbiomes of healthy (*n* = 8) and wheezing (*n* = 17) children. Community assembly mechanisms were inferred using β-nearest taxon index (βNTI) and Raup–Crick Bray–Curtis (RCbray) metrics. Thresholds of |βNTI|= 2 and |RCbray|= 0.95 are indicated by horizontal and vertical dashed lines, respectively

To investigate processes that may shape nasal and stool bacterial communities, we estimated the relative contribution of stochastic and deterministic assembly mechanisms using βNTI and RCbray metrics (Fig. 3). Stochastic processes dominated the classifications in both compartments. Whereas HC nasal communities were classified mainly as dispersal limitation (restricted microbial exchange between individuals/environments) or drift (random fluctuations in microbial abundances), a small fraction of pairwise comparisons in wheezers was also assigned to variable selection. Stool communities were also classified mainly as dispersal limitation, with a larger contribution of selective processes among wheezers than among HC. Given the small group sizes and cross-sectional design, these patterns are hypothesis-generating and do not identify the responsible host or environmental mechanisms.

**Fig. 4:**
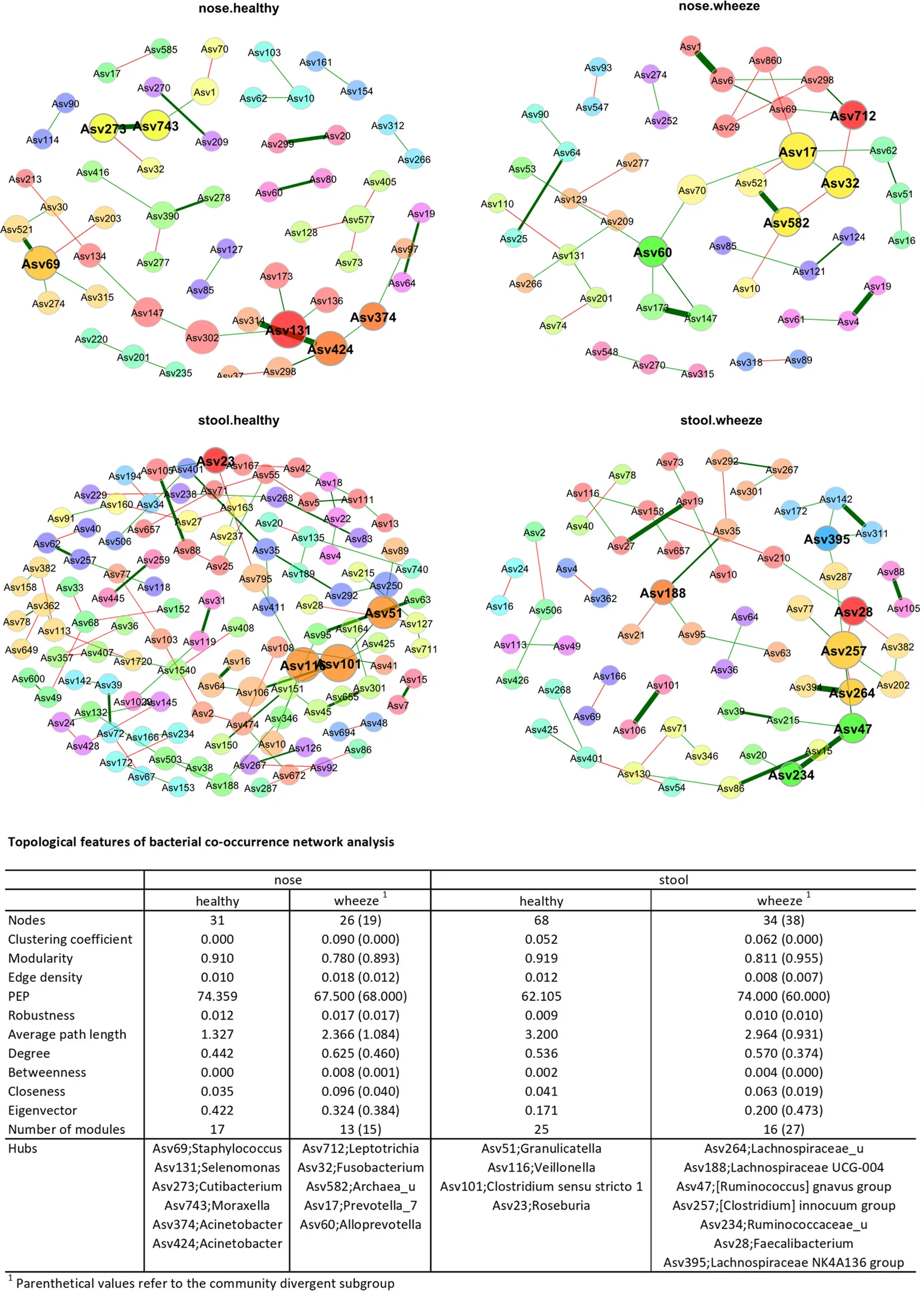
Exploratory bacterial co-occurrence networks in healthy (n=8) and wheezing (n=17) children. Bacterial co-occurrence networks were constructed from core ASV, defined as ASV present in ≥ 50% of subjects, in nasal and stool samples from healthy and wheezing children. Nodes represent ASV and edges represent inferred associations between pairs of ASV estimated using SpiecEasi (green: positive associations; red: negative associations). Modules, shown in different colours, represent groups of highly interconnected nodes. Hub taxa are highlighted in bold, and node size is proportional to eigenvector centrality. The accompanying table summarises key network topological characteristics, including the number of nodes, clustering coefficient, modularity, positive edge percentage (PEP), edge density, robustness, average path length, centrality measures (degree, betweenness, closeness and eigenvector centrality), and the number of modules

Exploratory co-occurrence networks constructed separately for HC and wheezers showed different topological summaries in nasal and stool bacteriomes (Fig. 4). Networks estimated for wheezers had fewer nodes and modules and lower modularity in both compartments, while clustering coefficients and several centrality measures were higher. The distance-defined divergent subgroup showed higher modularity and lower clustering and centrality measures than the overall wheezer group. Network hubs also differed descriptively between groups: in nasal samples, hub ASV shifted from *Staphylococcus* and *Cutibacterium* in HC to *Leptotrichia*, *Fusobacterium*, and *Prevotella* spp. in wheezers; in stool samples, hubs shifted from *Veillonella* and *Roseburia* toward Lachnospiraceae and *Ruminococcus*. These separately estimated networks were not subjected to inferential testing of between-group differences and therefore do not demonstrate altered microbial interactions or ecosystem fragmentation.

### Exploratory cross-compartment correlations between nasal and stool bacterial communities

**Fig. 5:**
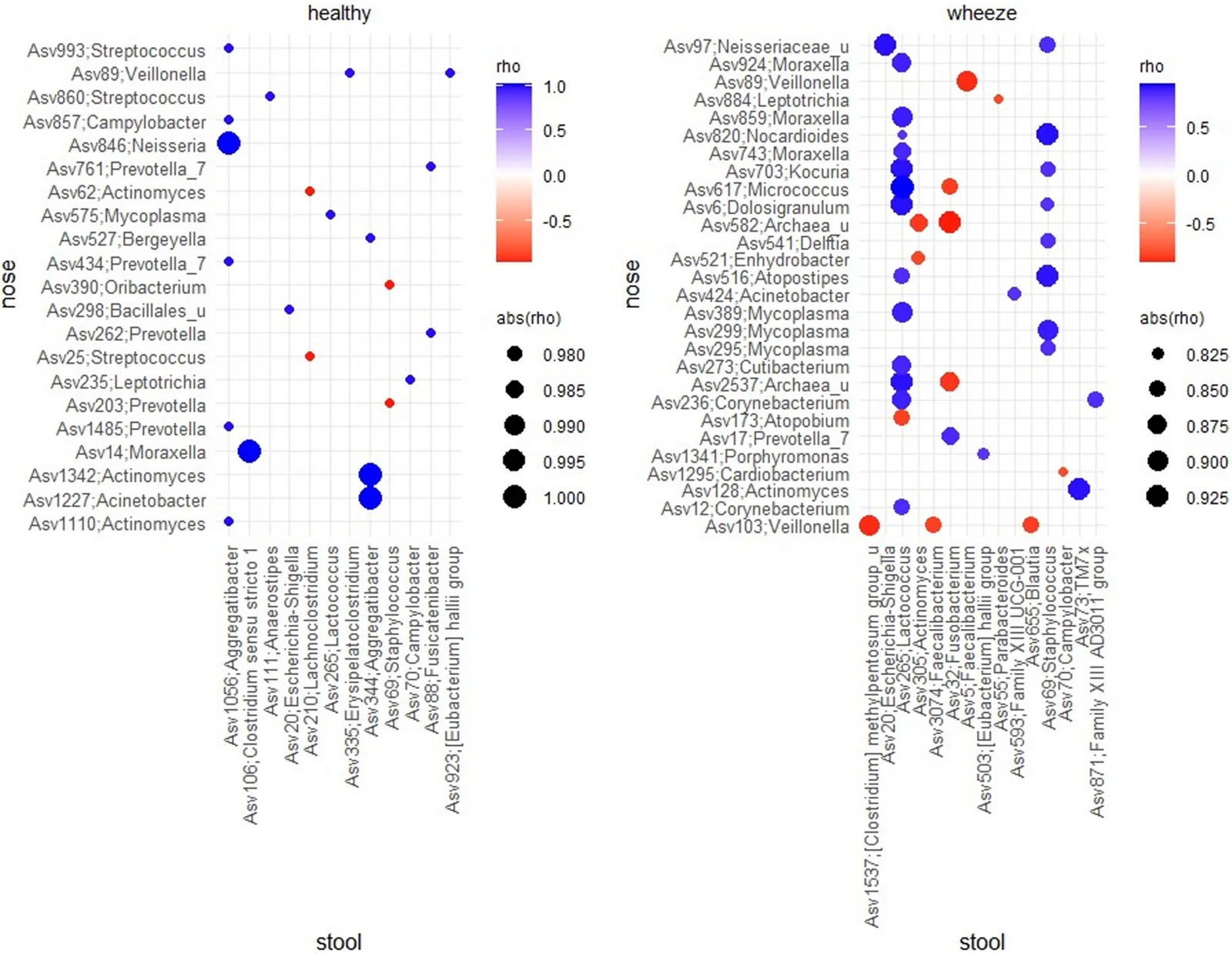
Within-group cross-compartment correlations in healthy (n=8) and wheezing (n=17) children. Spearman correlations were calculated between individual ASV in nasal and stool samples. Only correlations passing the Benjamini–Hochberg-adjusted threshold (p<0.05) are shown. Correlation coefficients were not compared directly between groups

Within-group Spearman analyses identified different sets of nasal–stool correlations that passed the multiple-testing threshold (Fig. 5): 42 correlations in HC (|ρ|≥0.97) and 53 in wheezers (|ρ|≥0.82), most of which were positive. The correlation coefficients were not compared directly between groups; therefore, the counts should be interpreted descriptively and in light of the unequal group sizes.

In HC, positive correlations were mainly observed among commensals, including nasal ASV assigned to *Prevotella*, *Neisseria*, *Actinomyces*, and *Veillonella* and stool ASV related to *Aggregatibacter*. In addition, stool ASV210 (*Lachnoclostridium)* and ASV69 (*Staphylococcus*) were negatively associated with nasal ASV assigned to *Actinomyces, Streptococcus*, and *Prevotella*, suggesting coordinated variation across compartments in HC.

Among wheezers, several nasal ASV assigned to *Corynebacterium*, *Dolosigranulum*, and *Moraxella* correlated positively with stool *Lactococcus* ASV265 (ρ=0.82–0.94). Further within-group correlations included nasal *Dolosigranulum* and *Mycoplasma* ASV with stool *Staphylococcus* (ASV69), nasal *Veillonella* with stool *Faecalibacterium* and *Blautia*, and stool *Escherichia–Shigella* (ASV20) with nasal *Neisseriaceae*_u (ASV97). The distance-defined divergent subgroup retained fewer statistically supported cross-compartment correlations, mainly involving stool *Escherichia–Shigella*, *Faecalibacterium*, and Rothia (Supplementary Fig. S4). These analyses identify co-variation within groups but do not establish that correlation patterns differed statistically between groups.

## Discussion

### Nasal bacterial communities show heterogeneity and exploratory Moraxella-associated patterns

In this cross-sectional exploratory pilot study, we analysed paired nasal and stool bacterial communities from preschool children with recurrent wheeze and HC. Overall richness and global community composition were comparable between groups, while nasal communities among wheezers showed greater inter-individual dispersion of borderline statistical support. The main results therefore concern heterogeneity of nasal profiles rather than a consistent group-wide microbial signature.

*Moraxella* is a common colonizer of the upper airways, and previous studies have reported contrasting associations with respiratory health. It can occur as part of healthy early-life communities [10, 24], but *Moraxella*-dominated profiles have also been associated with respiratory infection and wheezing [11, 25]. In the present dataset, *Moraxella* abundance did not differ significantly between the full wheezer and HC groups. *Moraxella*-rich profiles co-occurred with lower richness and evenness only within an exploratory distance-defined subgroup. This observation does not establish *Moraxella* as a driver and may reflect prior viral infection, treatment exposure, host immunity, or another unmeasured factor. Larger longitudinal studies stratified by infection and treatment history, with metagenomic, metabolomic, and immunological measurements, will be needed to test these alternatives.

### Stool bacterial communities show limited differences at preschool age

Stool bacterial communities showed no statistically supported genus-level differences between wheezers and HC. This result is compatible with previous reports that intestinal microbial signatures associated with asthma risk may be strongest during early infancy [15, 17, 18], but the present cross-sectional study cannot determine whether earlier differences normalized, persisted below the detection limit, or were absent in these children.

Longitudinal studies have identified infancy as an important period for intestinal microbial development and immune maturation [14, 16]. By preschool age, diet and environmental exposures may contribute to convergence of stool bacterial communities. Antibiotic exposure may also influence these patterns [26, 27], but the high proportion of missing antibiotic data in the present cohort precluded an informative assessment. The small sample size further limited power to detect subtle associations. Larger longitudinal cohorts are required to determine how intestinal profiles relate to recurrent wheeze, asthma development, systemic metabolites, and immune phenotypes.

### Exploratory ecological and cross-compartment patterns

Beyond taxonomic composition, the ecological analyses yielded descriptive differences in community-assembly classifications between HC and wheezers. Stochastic processes dominated both compartments, consistent with models in which dispersal and drift contribute to microbial colonization [28]. The larger descriptive contribution of variable selection among wheezers may motivate hypotheses involving inflammation, prior viral infection, treatment, or local immune responses, but the present analysis cannot identify the responsible mechanism. The separately estimated co-occurrence networks also differed in modularity, node number, and centrality. Although reduced network complexity has been linked to lower ecological resilience in other systems [29, 30], association networks derived from 16S rRNA gene data do not measure metabolic interaction, cross-feeding, stability, or resilience directly. Differences in network topology may also arise from sample size, prevalence filtering, and compositional data structure. Consequently, these results should be treated as hypotheses for future studies integrating metagenomics, metabolomics, culture-based interaction experiments, and immune profiling rather than as evidence of ecosystem fragmentation.

Finally, different sets of cross-compartment correlations passed the multiple-testing threshold in HC and wheezers. Such co-variation may reflect shared diet, age, medication, infection history, immunity, or other environmental exposures. It neither demonstrates direct microbial exchange between the gut and nose nor establishes a gut-to-airway mechanism. Functional links through microbial metabolites, including short-chain fatty acids [31, 32], remain biologically plausible but were not measured in this study.

This study has several important limitations. The sample size was small, the design was cross-sectional, and convenience sampling may have introduced selection bias. Nasal swabs sample the nasal upper-airway niche and cannot be treated as direct measurements of the lower-airway microbiome. Because nasal swabs are low-biomass samples, conclusions also depend on quantitative evaluation of negative controls and potential batch effects. Although sequencing depth in the blank controls was very low, low-level background DNA was detected, and the possibility of low-level contamination cannot therefore be completely excluded, particularly for ASV detected at very low abundance in biological samples. However, no ASV was recurrent across blank controls, and only one ASV was identified as a potential contaminant by decontam and subsequently removed. Prior RSV infection was reported in 7 of 15 wheezers with available data but in none of the 7 controls with available data, and systemic or inhaled steroid exposure occurred only in the wheezer group. Effects of wheeze therefore cannot be separated from prior infection or treatment in this dataset, and sensitivity analyses are constrained by the remaining sample size. In addition, 16S rRNA gene metabarcoding has limited taxonomic resolution and does not establish strain-level identity or functional potential. Larger longitudinal studies incorporating quantitative contamination control, metagenomics, metabolomics, and immune profiling are needed to validate and extend these observations.

## Conclusions

In this exploratory pilot study, overall richness and global community composition did not differ significantly between preschool children with recurrent wheeze and healthy controls. Wheezers showed greater heterogeneity of nasal bacterial profiles, and an exploratory distance-defined subgroup contained *Moraxella*-rich profiles with lower diversity. These findings do not establish *Moraxella*-driven clustering or causality. Group-specific community-assembly classifications, network summaries, and cross-compartment correlation sets provide hypotheses for future work but require independent validation in larger longitudinal cohorts with functional measurements.

## Supplementary Information

Below is the link to the electronic supplementary material.

Supplementary file1 (PDF 1.38 MB)

## Data Availability

The sequence data generated and analyzed during this study have been deposited in the NCBI Sequence Read Archive (SRA) under BioProject accession number PRJNA1499410 (reviewer link: https://dataview.ncbi.nlm.nih.gov/object/PRJNA1499410?reviewer=okpdiae11ucclqmiq09i436qvh). Associated sample metadata are not publicly available in order to protect participant privacy but may be obtained from the corresponding author upon reasonable request.
