# Supplementary material for: Exploratory analysis of nasal and gut microbial community structure in preschool children with recurrent wheeze
Source: Microb Ecol. 2026 Sep 1;89:196. doi: 10.1007/s00248-026-02867-3 (PMC13719913; doi:10.1007/s00248-026-02867-3)
Supplement: Supplementary file 1 [file 248_2026_2867_MOESM1_ESM.pdf]

Supplementary Material

Supplementary Methods

Study design and procedures

The study design and procedures have been described in detail previously [1, 2]. For easier access we included this information here:

The ALLIANCE cohort is recruited at five pediatric centers (Cologne, Hannover, Lubeck, Marburg, Munich) and two adult centers (LungenClinic Grosshansdorf, Research Centre Borstel), all affiliated with the German Center for Lung Research (DZL). Recruitment started in 2013. Participants with preschool wheeze and asthma had annual study visits while healthy controls were assessed only once. Visits were postponed in case of upper respiratory tract infections, asthma exacerbations (adults) or fever >38.5°C in the past two weeks (children).

Caregivers and adult participants completed questionnaires covering demographic factors, early-life exposures, respiratory symptoms, medical history and medication use. Recorded variables included age, sex, BMI, mode of delivery, breastfeeding, siblings, pet contact (cat/dog), childcare, wheeze and cough during the previous 12 months, previous RSV infection, physician-diagnosed asthma, hay fever, eczema or food allergy, and use of hypoallergenic formula, antibiotics for recurrent cough, or inhaled/systemic corticosteroids. Definitions of clinical and lifestyle variables have been described previously [1]. Questionnaire-based outcomes were defined as follows: wheeze episode as salbutamol use due to wheeze on >2 of 7 days; exacerbation as a wheeze episode requiring systemic corticosteroids or hospitalization; and asthma control according to GINA guidelines.

Differential blood counts and total IgE were measured in routine on-site hospital laboratories. Allergen-specific IgE was assessed centrally using Euroline™ (Euroimmun, Germany) against a panel of common aeroallergens and food allergens [2]. Atopy was defined as ≥1 allergen-specific IgE ≥0.7 kU/L, based on previous findings from the ALLIANCE cohort [3]. Elevated blood eosinophil counts were defined as ≥470 cells/µl, corresponding to the 90th percentile of healthy controls in the ALLIANCE cohort.

DNA extraction and library preparation

DNA of nasal swabs was extracted via freezing-heating cycles (5 min, 3 times) following ultrasonication (S220 Focused-ultrasonicator Covaris) with 6 cycles of 5 s at peak power=200/duty factor=2, 30 s peak power=275/duty factor=5 at 6°C [4]. DNA of stool samples was extracted from 0.4 g feces material using the phenol-chloroform-based protocol described by Lueders et al. [5] and the Precellys24 Instrument (PeqLab, Erlangen, Germany).

Amplicon sequencing of the V4 hypervariable region of the 16S rRNA gene was performed on a MiSeq Illumina instrument (MiSeq Reagent Kit v3 (600 Cycle); Illumina, San Diego, CA, USA) using the universal eubacterial primers 515F [6] and 806R [7]. PCR was done using NEBNext high fidelity polymerase (New England Biolabs, Ipswich, USA) in a total volume of 25 µl (10 ng DNA template (nose) / 5 ng DNA template (stool), 12.5 µl polymerase, 5 pmol of each primer, 2.5 µl 3% BSA) and the following PCR conditions: 5 min at 98˚C; 30 cycles (nose) / 25 cycles (stool) of 10 s at 98˚C, 30 s at 55 ˚C, 30 s at 72 ˚C; 5 min 72 ˚C. To identify potential contaminants deriving from DNA extraction and library preparation, both extraction and PCR no template control samples (each 4) were processed alongside biological samples. PCR products were purified using MagSi NGSprep Plus beads (Steinbrenner, Wiesenbach, Germany) and quantified via PicoGreen assay. Subsequently, indexing PCR was performed using the Nextera XT Index Kit v2 (Illumina, Inc. San Diego, CA, US) in a total volume of 25 µl (10 ng DNA template, 12.5 µl NEBNext high fidelity polymerase, 2.5 µl of each indexing primer) and the following PCR conditions: 30s at 98 ˚C; 8 cycles of 10 s at 98 ˚C, 30s at 55 ˚C, 30s at 72 ˚C; 5min 72 ˚C. Indexing PCR products were purified using MagSi NGSprep Plus beads, qualified and quantified via a Fragment Analyzer™ instrument (Advanced Analytical Technologies, Inc., Ankeny, USA) and pooled in an equimolar ratio of 4nM. All biological and control samples were sequenced in a single sequencing run to avoid batch effects related to processing steps. Sequence data generated in this study have been deposited in the NCBI Sequence Read Archive under BioProject PRJNA1499410.

Sequence processing

FASTQ files were trimmed with a minimum read length of 50 using Cutadapt [8] and quality control was performed via FastQC [9]. For subsequent data analysis, the DADA2 pipeline v 1.30 [10] was used with the following trimming and filtering parameters: 20 bp were removed n-terminally and reads were truncated at position 240 (forward) and 200 (reverse), respectively, with expected error of 3 (forward) and 4 (reverse). Taxonomic analysis was performed using SILVA v138.1. Reads were excluded if classified as mitochondria or chloroplast or if the phylum was missing. All blank controls were processed

and sequenced alongside the biological samples. After DADA2 denoising, blank controls yielded 0–6 reads per sample, corresponding to <0.01% of the mean sequencing depth of the biological samples (74,896 reads). Across all blank controls, seven amplicon sequence variants (ASV) were detected and assigned to the genera *Agathobacter, Bacteroides, Faecalibacterium, Moraxella, Pediococcus*, and *Streptococcus*, while one ASV remained unclassified at the phylum level. None of these ASV was detected in more than one blank control. Only ASV 653 (*Pediococcus*), was consistently identified as a potential contaminant using prevalence-based decontam analysis at thresholds of 0.1 and 0.5 and was therefore removed from all biological samples. Quality-control information for the blank controls, including DNA concentration, read numbers before and after DADA2 denoising, detected ASV, their occurrence in biological samples, and their mean relative abundance, is provided in Supplementary Table S1. Given the extremely low sequencing depth of the blank controls, the absence of recurrent ASV across blank controls, and the decontam results, there was no evidence of systematic contamination by the remaining ASV, which were therefore retained in the biological-sample dataset. Following removal of ASV 653, the dataset comprised 3,744,773 reads assigned to 2,177 ASVs for subsequent analyses.

Statistical analysis

All plots and statistics were performed in R version 4.5.1 (https://www.R-project.org). Prior to the main analysis, potential covariates (age, gender, cough antibiotics, siblings, daycare, and pet contact) were tested individually for association with microbiome outcomes due to the modest sample size (n = 25). Covariates improving model fit, as judged by metrics like delta AIC, beta coefficients and CI for mixed-effect models or explained variance for PERMANOVA, were included in the final analysis.

Alpha diversity was calculated using species richness, evenness as well as Shannon diversity index. Associations with covariates were assessed using generalized linear mixed-effects models (R package glmmTMB), including study subjects as a random effect. Covariates improving model fit in pre-tests (age and gender) were retained. Analyses were performed on TMM-normalized data [11] and raw counts, with log-transformed sequencing depth as covariate for raw counts when it was significantly correlated with the response variable (assessed using cor.test). Beta diversity was analyzed using unweighted and weighted UniFrac distances on raw counts, as well as Aitchison distance on CLR-transformed data. Associations with covariates were tested via PERMANOVA, with permutations restricted within study subjects using the strata option (R package vegan). Based on pre-tests, age and gender was included

as covariate. For both alpha and beta diversity analysis, p value adjustment for multiple comparisons was performed with Benjamini-Hochberg correction.

To identify microbial taxa differing between healthy and wheezing children, ANCOM-BC2 (Analysis of Compositions of Microbiomes with Bias Correction 2) was used [12], with age and gender as covariates. Phylogenetic trees for the ASV of interest were constructed including SILVA-curated reference sequences (https://www.arb-silva.de/). Sequences were aligned with ClustalW following tree optimization via the Maximum Likelihood method (GTR model) and 1000 bootstrap iterations for reliability (R packages ape, phangorn, msa).

For analysis of within-group variation, the average weighted UniFrac distance between each subject and the group centroid was calculated (beta-dispersion). Statistical analysis was performed like for alpha diversity via generalized linear mixed-effects models (R package glmmTMB) and Benjamini-Hochberg p value correction for multiple comparisons, including study subjects as a random effect and age and gender as covariates. In addition, Levene's Test was used to test for equality of variances within the groups.

To search for associations between individual variability (beta-dispersion), demographic/lifestyle variables, hematological immune markers (hemoglobin, thrombo-, leuko-, lymphocytes, neutro-, eosino-, basophils, immunoglobulin E), and microbial taxa, correlation analyses were performed, with Pearson correlations for numerical variables and Spearman correlations for the comparison of numeric and microbiome data after CLR-transformation. P value adjustment for multiple comparisons was performed with Benjamini-Hochberg correction.

The core microbiome per group was defined as ASV or genera present in at least 80 % of subjects without setting an additional abundance cutoff.

To explore heterogeneity among wheezing children, samples were classified as healthy-like or community-divergent based on their microbiome's distance to the centroid of healthy samples. First, Bray-Curtis distances were calculated on TSS-normalized data, and then Euclidean distances were computed from each sample to the healthy centroid in the ordination space. Wheezing samples within the 90th percentile of the healthy centroid distances were considered healthy-like, while those exceeding this threshold were classified as community-divergent. This resulted in body site-specific subgroup sizes of 9 and 8 for nasal samples and 10 and 7 for stool samples (healthy-like and community-divergent, respectively). Selected variables were fitted to the ordination space as vectors using the envfit function (R package vegan), with significance assessed via permutation tests. Further statistical analysis

between those subgroups were performed as described above using generalized linear mixed-effects models (R package glmmTMB), ANCOM-BC2 and correlation analyses.

Microbial community assembly was assessed using the β-nearest taxon index (βNTI) and Raup-Crick–based Bray-Curtis (RCbray) approach following Stegen et al. [13]. Although βNTI was originally developed for macroecological systems, it is widely applied in microbial ecology because it quantifies phylogenetic turnover relative to null expectations, enabling inference of deterministic versus stochastic processes. Phylogenetic relatedness serves as a proxy for ecological similarity due to trait conservation in microbes. This framework was applied to evaluate how deterministic and stochastic processes shape microbial communities across healthy and wheezing children, where wheezing may impose strong selective pressures. For βNTI, thresholds of ±2 were used to define deterministic selection, further categorized as heterogeneous/variable selection (βNTI > +2; divergent community configurations) and homogeneous selection (βNTI < −2; convergence toward similar composition). For pairwise comparisons with |βNTI| < 2, RCbray distinguished stochastic processes: RCbray > +0.95 indicated dispersal limitation, RCbray < −0.95 indicated homogenizing dispersal, and |RCbray| ≤ 0.95 indicated undominated processes. Analyses were performed in R using the *picante* and *vegan* packages with null distributions generated from 999 randomizations. A phylogenetic tree was constructed prior to these analyses by aligning sequences with MAFFT (FFT-NS algorithm) and building the tree with FastTree under the GTR+CAT substitution model. The *ape* package was used to handle phylogenetic distances via a *phyloseq* object.

Microbial co-occurrence networks were constructed using the R package NetCoMi v1.1.0 [14], applying the “signed” transformation to convert estimated associations into dissimilarities and including only ASV present in ≥50% of samples per group. Networks were inferred with the SpiecEasi framework on CLR-transformed data, with zero replacement via multiplicative imputation and the Meinshausen-Bühlmann neighborhood selection method for network inference. Network topology was characterized by modular structure (fast-greedy clustering) and identification of hub taxa based on degree and eigenvector centrality (top 10%). Centrality measures (degree, betweenness, closeness, eigenvector) were normalized, and weighted node degrees were calculated to assess connectivity patterns across microbial communities.

To evaluate cross-compartment associations between nasal and stool microbiomes, raw counts were transformed to relative abundances and CLR-transformed. Spearman correlations between all nose-stool pairs were calculated using the rcorr function (R package Hmisc), with p-values adjusted via

Benjamini-Hochberg. Significant correlations ($p < 0.05$) were visualized as a bipartite network (R packages igraph, ggraph), with nodes representing taxa and edges indicating significant associations. If not stated otherwise, all plots were created in R using ggplot2 [15], ggpubr [16], and eulerr [17].

Supplementary References

1. Fuchs O, Bahmer T, Weckmann M, et al. The all age asthma cohort (ALLIANCE) - from early beginnings to chronic disease: a longitudinal cohort study. BMC Pulmonary Medicine. 2018;18(1):140.
2. Maison N, Omony J, Illi S, et al. T2-high asthma phenotypes across lifespan. Eur Respir J. 2022;60(3):2102288.
3. Skevaki C, Tafo P, Eiringhaus K, et al. Allergen extract- and component-based diagnostics in children of the ALLIANCE asthma cohort. Clinical & Experimental Allergy. 2021;51(10):1331-45.
4. Pust M-M, Wiehlmann L, Davenport C, et al. The human respiratory tract microbial community structures in healthy and cystic fibrosis infants. npj Biofilms and Microbiomes. 2020;6(1):61.
5. Lueders T, Manefield M, Friedrich MW. Enhanced sensitivity of DNA- and rRNA-based stable isotope probing by fractionation and quantitative analysis of isopycnic centrifugation gradients. Environ Microbiol. 2004;6(1):73-8.
6. Parada AE, Needham DM, Fuhrman JA. Every base matters: assessing small subunit rRNA primers for marine microbiomes with mock communities, time series and global field samples. Environ Microbiol. 2016;18(5):1403-14.
7. Apprill A, McNally S, Parsons R, et al. Minor revision to V4 region SSU rRNA 806R gene primer greatly increases detection of SAR11 bacterioplankton. Aquat Microb Ecol. 2015;75.
8. Martin M. Cutadapt removes adapter sequences from high-throughput sequencing reads. EMBnetjournal. 2011;17:10-2.
9. Andrews S. FastQC: A Quality Control Tool for High Throughput Sequence Data. https://wwwbioinformaticsbabrahamacuk/projects/fastqc/. 2010.
10. Callahan BJ, McMurdie PJ, Rosen MJ, et al. DADA2: High-resolution sample inference from Illumina amplicon data. Nat Methods. 2016;13(7):581-3.
11. Robinson MD, McCarthy DJ, Smyth GK. edgeR: a Bioconductor package for differential expression analysis of digital gene expression data. Bioinformatics. 2009;26(1):139-40.
12. Lin H, Peddada SD. Multigroup analysis of compositions of microbiomes with covariate adjustments and repeated measures. Nat Methods. 2024;21(1):83-91.
13. Stegen JC, Lin X, Konopka AE, et al. Stochastic and deterministic assembly processes in subsurface microbial communities. ISME J. 2012;6(9):1653-64.
14. Peschel S. NetCoMi: Network Construction and Comparison for Microbiome Data. https://netcomide. 2025;R package version 1.2.0.
15. Wickham H. ggplot2: Elegant Graphics for Data Analysis. https://ggplot2tidyverseorg. 2016.
16. Kassambara A. ggpubr: 'ggplot2' Based Publication Ready Plots. R package version 0.6.0. https://rpkgsdatanoviacom/ggpubr/. 2023.
17. Larsson JG, Peter. A Case Study in Fitting Area-Proportional Euler Diagrams with Ellipses using eulerr. Proceedings of International Workshop on Set Visualization and Reasoning (SetVR 2018) co-located with 10th International Conference on the Theory and Application of Diagrams (Diagrams 2018), Edinburgh, UK, June 18, 2018 (pp 84-91). 2018;CEUR-WS.org. https://ceur-ws.org/Vol-2116/paper7.pdf.

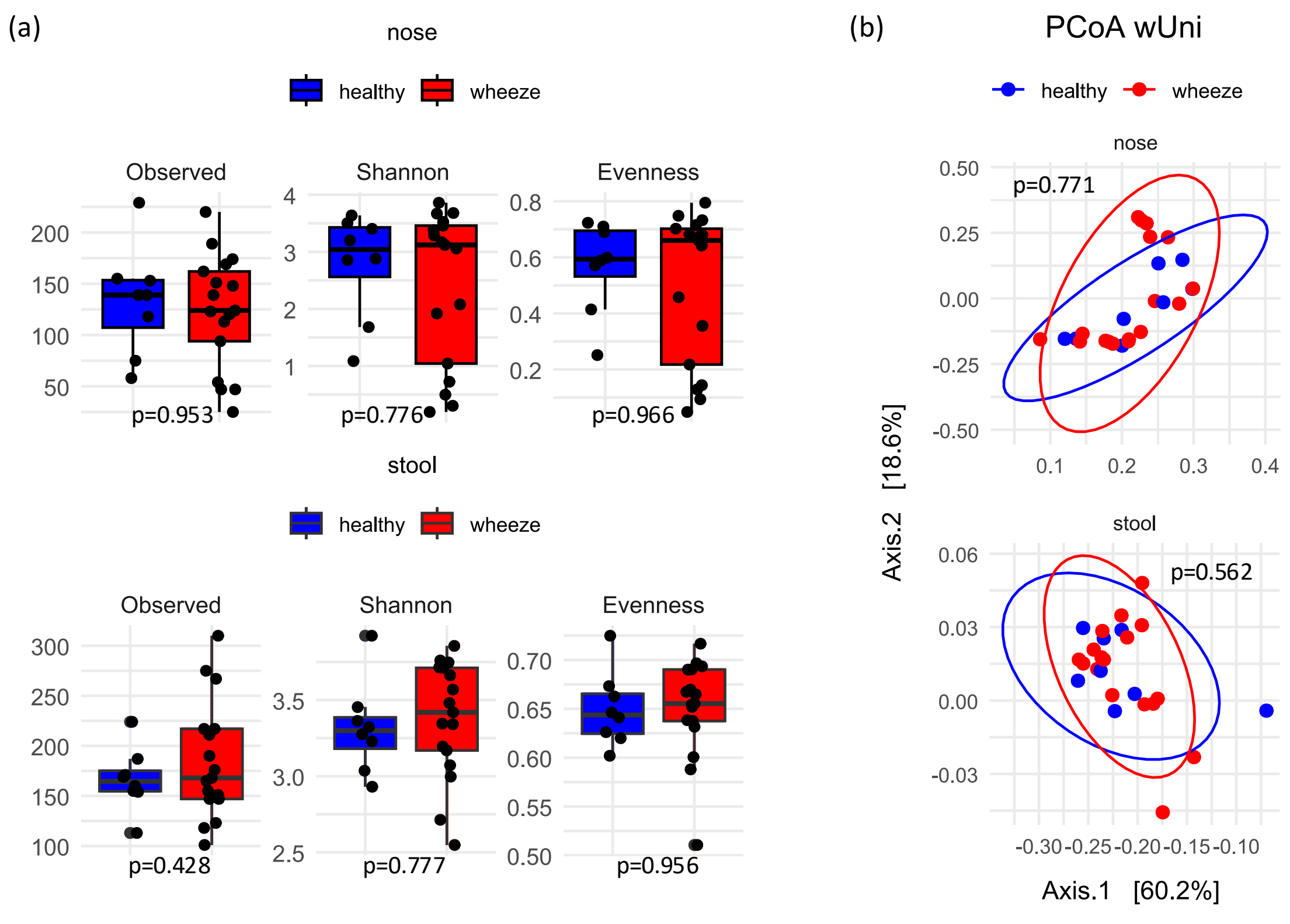

(c)

Measure	Material	Beta	CI_low	CI_high	p_value	p_adjusted	comparison
Observed	stool	-0.044	0.892	1.026	0.214	0.967	healthy vs wheeze
Observed	nose	-0.002	0.923	1.078	0.954	0.967	healthy vs wheeze
Shannon	stool	-0.034	-0.262	0.194	0.772	0.967	healthy vs wheeze
Shannon	nose	-0.145	-1.148	0.858	0.777	0.967	healthy vs wheeze
Evenness	stool	-0.003	-0.154	0.147	0.967	0.967	healthy vs wheeze
Evenness	nose	-0.214	-0.995	0.567	0.591	0.967	healthy vs wheeze

Measure	Material	R2	F	p_value	p_adjusted	comparison
wUni	nose	0.018	0.410	0.771	0.771	healthy vs wheeze
wUni	stool	0.043	1.130	0.316	0.562	healthy vs wheeze

Figure S1. Microbial diversity and overall community composition in nose and stool of healthy (n=8) and wheezing (n=17) children.

(a) Boxplots of alpha diversity measures (observed ASV, Shannon index and evenness). Group differences were assessed using generalized linear mixed-effects models with Benjamini–Hochberg adjustment. Effect sizes, 95% CI, and p values are gi

(b) Principal coordinates analysis (PCoA) of weighted UniFrac distances. Group differences were assessed using PERMANOVA with Benjamini–Hochberg correction.

(c) Results of statistical analysis. For alpha diversity, estimated group differences (beta), 95% confidence intervals, and p values were reported (top), whereas for beta diversity PERMANOVA R2 as the effect size, along with the pseudo-F statistic and permutation-derived p-values were shown (bottom).

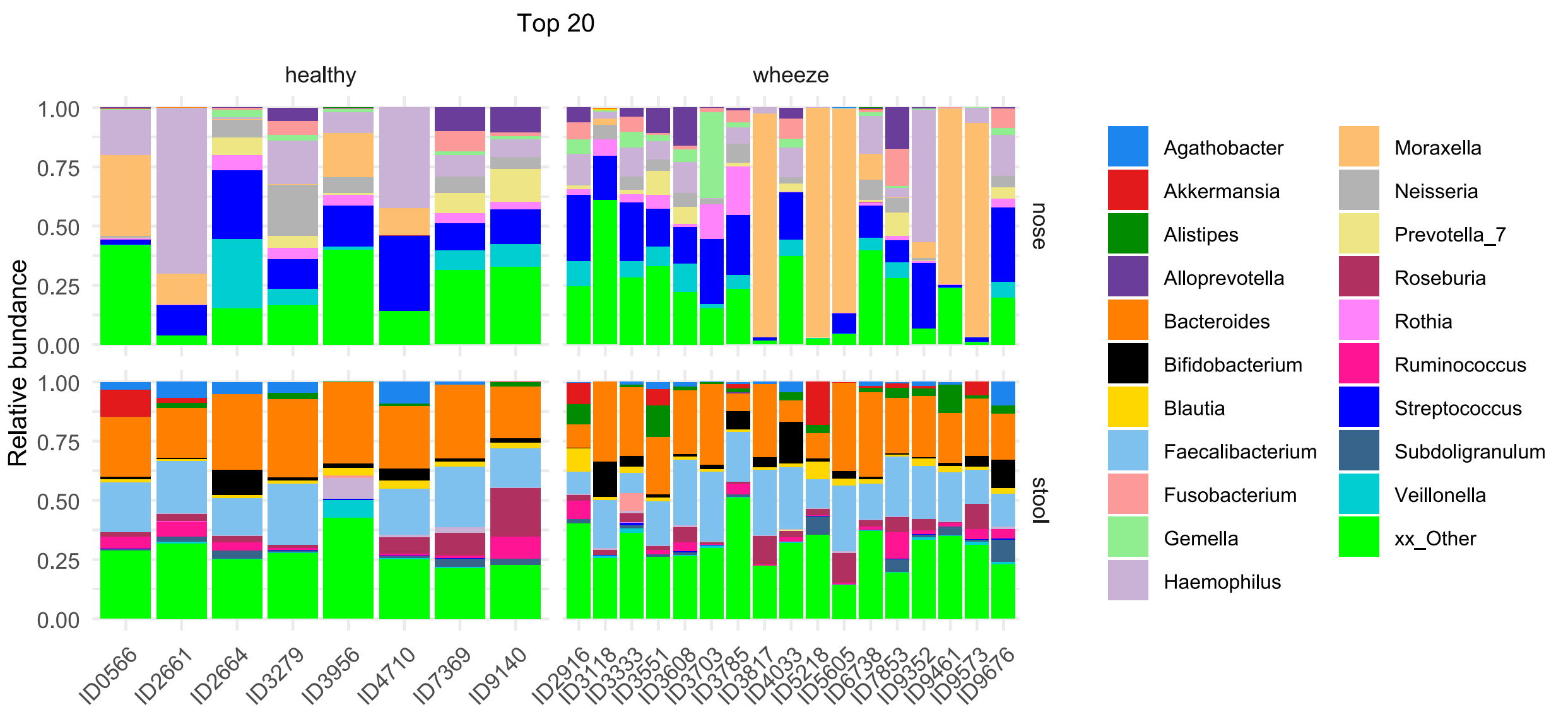

Figure S2. Relative abundance of the top 20 genera per subject

Supplementary Figure S2

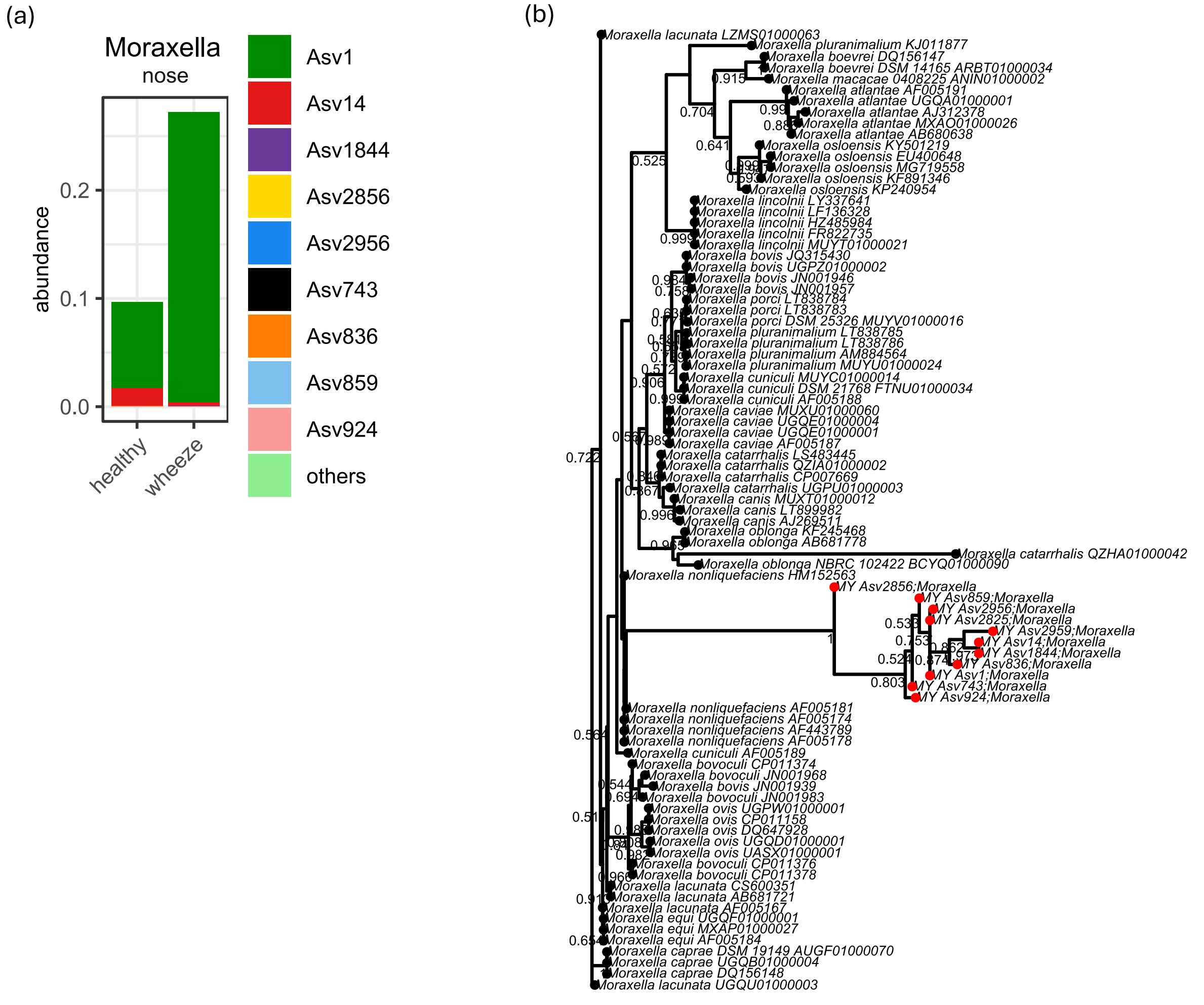

Figure S3. ASV assigned to the genus *Moraxella* identified in the study cohort.

(a) Relative abundance of *Moraxella*-ASV in healthy and wheezing children.

(b) Maximum-likelihood phylogenetic tree showing the placement of the detected Moraxella ASV (red dots) among reference sequences obtained from the SILVA database. Numbers at the nodes indicate bootstrap support values.

(a)

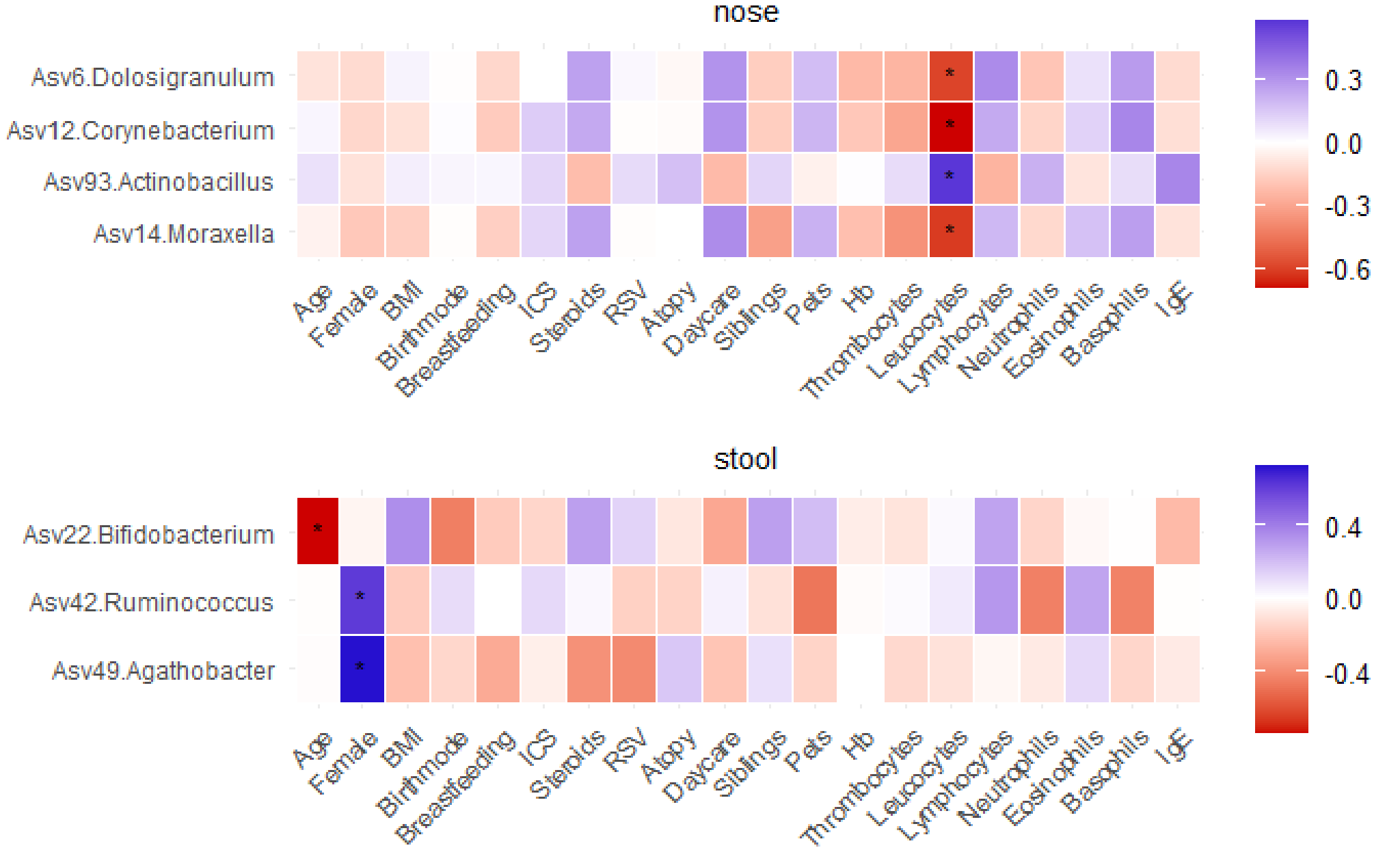

(b)

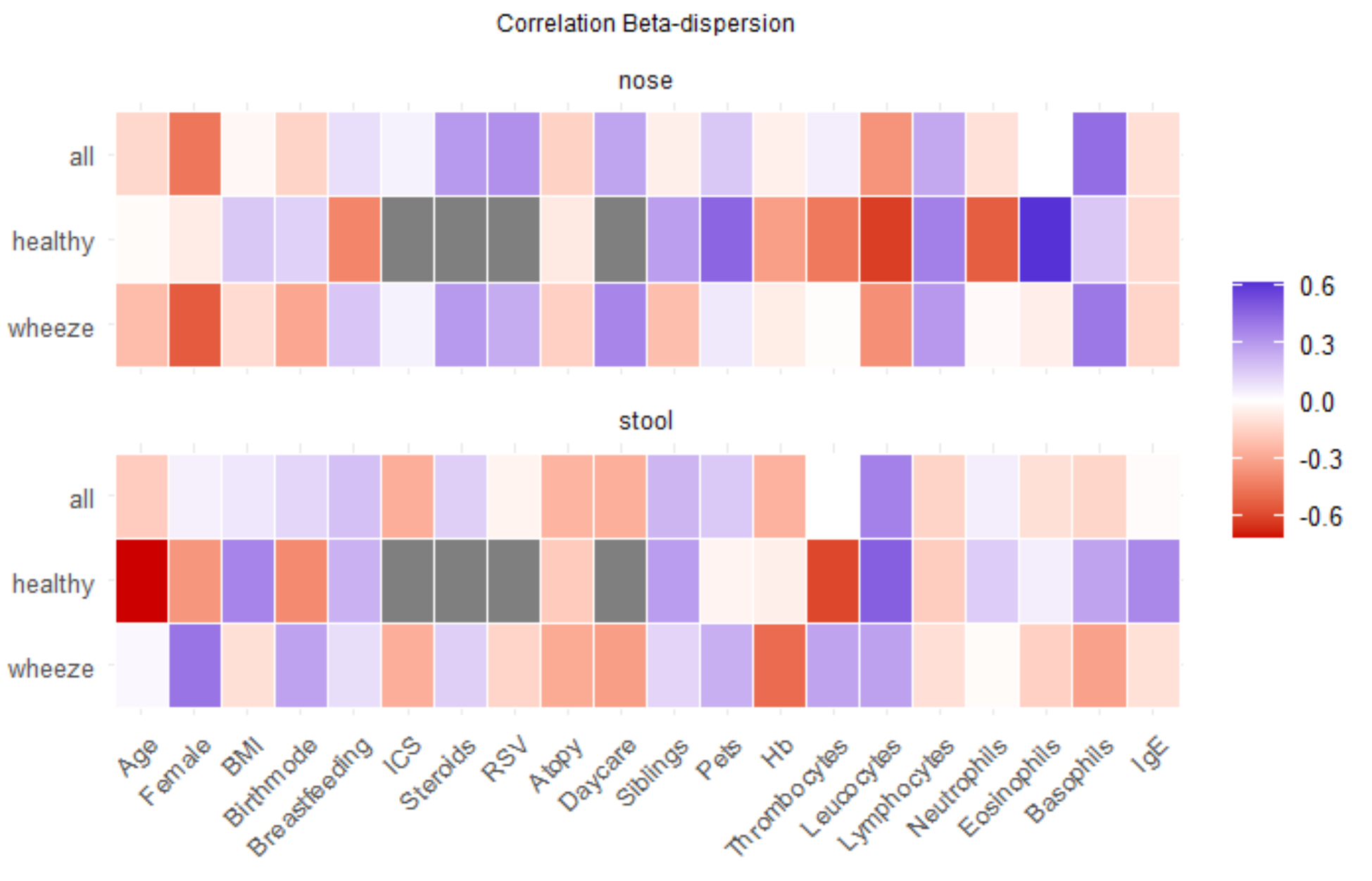

Figure S4. Associations between microbial community and demografic, lifestyle and immunological variables in nose and stool.

(a) Spearman correlations between individual ASV and selected demographic, lifestyle and immunological variables in nasal and stool samples. Asterisks indicate statistically significant correlations after Benjamini-Hochberg adjustment ($p<0.05$), calculated using cor.test.

(b) Pearson correlations between beta-dispersion and selected demographic, lifestyle and immunological factors in nasal and stool samples. Asterisks indicate statistically significant correlations after Benjamini-Hochberg adjustment ($p<0.05$), calculated using cor.test.

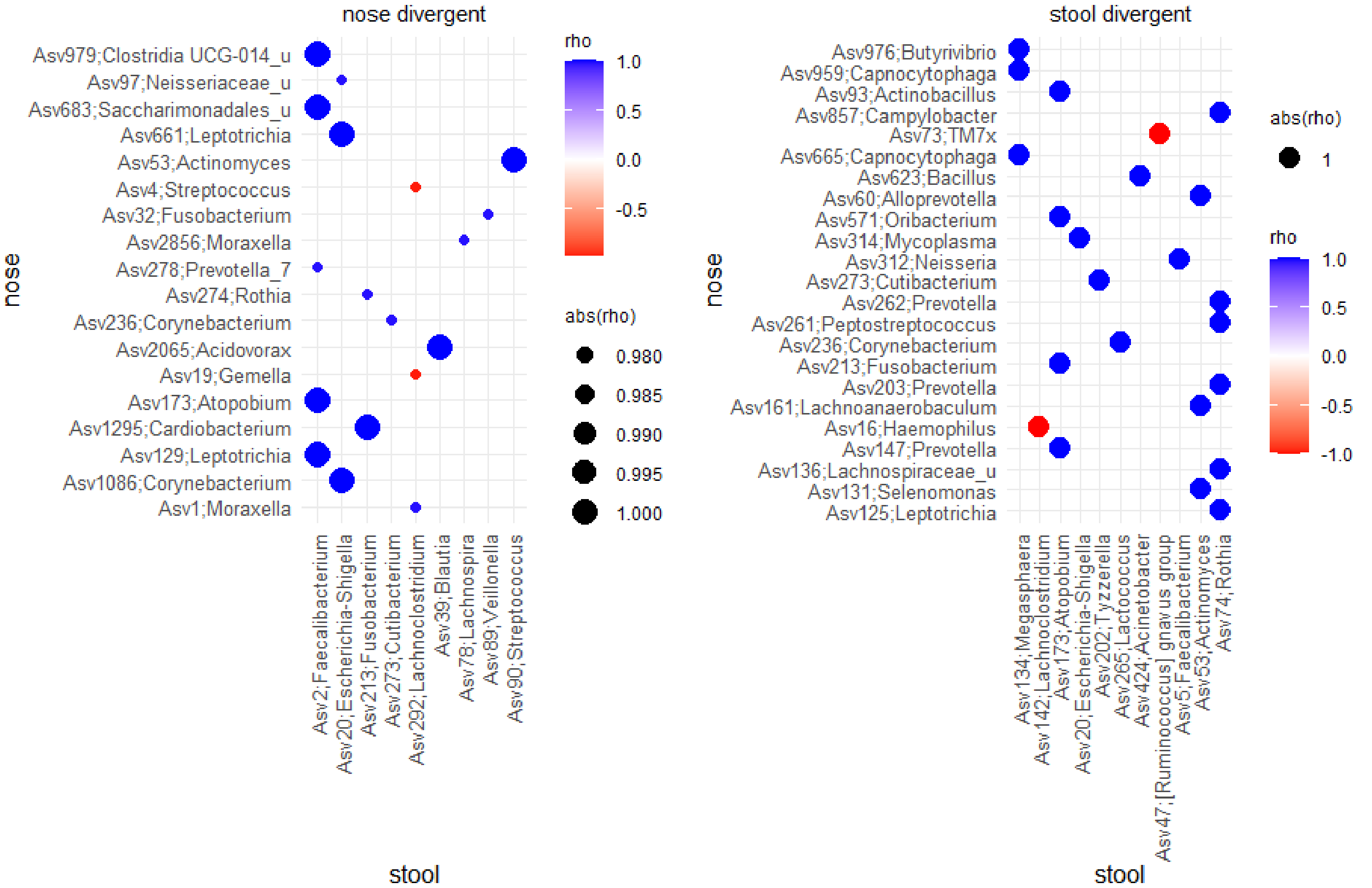

Figure S5. Pairwise cross-compartment correlations in community divergent subgroups. Spearman correlations were calculated between individual ASV in nasal (n=8) and stool (n=7) samples. Only statistically significant correlations after Benjamini–Hochberg adjustment ($p<0.05$) are shown.

Supplementary Figure S5

Supplementary Table S1. Quality control information of extraction blanks and PCR no template controls (each 4) performed alongside biological samples to identify potential contaminants deriving from DNA extraction and library preparation. All biological and control samples were sequenced in a single sequencing run to avoid batch effects related to processing steps.

Control ID	Control type	DNA concentration (ng/µL)	Reads after sequencing	Reads after DADA2	Number of ASV	Taxonomy of ASV	Biological samples positive	Mean relative abundance in biological samples (%)	Mean relative abundance among positive samples (%)	Identified by decontam
Ex1	Extraction blank stool	0.87	15	6	2	Bacteroides, Asv41<br>Agathobacter, Asv49	16<br>22	0.39<br>0.39	1.23<br>0.89	No<br>no
Ex2	Extraction blank stool	0.71	23	0	-	-	-	-	-	-
Ex3	Extraction blank nose	1.09	9	2	1	Pediococcus, Asv653	1	0.00	0.02	yes
Ex4	Extraction blank nose	0.30	107	0	-	-	-	-	-	-
PCR1	PCR blank	-	209	4	3	Moraxella, Asv1<br>Streptococcus, Asv11<br>Bacteria unclassified	19<br>10<br>- *	10.39<br>1.53<br>- *	27.33<br>7.64<br>- *	no<br>no<br>-
PCR2	PCR blank	-	7	2	1	Faecalibacterium, Asv5	27	3.45	6.38	no
PCR3	PCR blank	-	1	0	-	-	-	-	-	-
PCR4	PCR blank	-	67	0	-	-	-	-	-	-

* ASV lacking phylum-level annotation were removed from samples

Supplementary Table S2. Core ASV (≥80% prevalence within group) in nasal and stool samples, shown as binary presence/absence (1/0). Nose: 9 shared, 4 healthy-specific, 9 wheezer-specific ASV; stool: 30 shared, 11 healthy-specific, 13 wheezer-specific ASV.

	nose		stool		
Taxa	Healthy	Wheeze	Healthy	Wheeze	Prev
Asv1;Moraxella	1	0	0	0	prev0.8
Asv10;Veillonella	0	1	1	1	prev0.8
Asv101;Clostridium sensu stricto 1	0	0	1	0	prev0.8
Asv105;Monoglobus	0	0	1	1	prev0.8
Asv106;Clostridium sensu stricto 1	0	0	1	1	prev0.8
Asv110;Neisseria	0	1	0	0	prev0.8
Asv111;Anaerostipes	0	0	1	1	prev0.8
Asv114;Porphyromonas	1	1	0	0	prev0.8
Asv116;Veillonella	0	0	1	1	prev0.8
Asv13;Roseburia	0	0	1	0	prev0.8
Asv132;Bifidobacterium	0	0	0	1	prev0.8
Asv142;Lachnoclostridium	0	0	1	1	prev0.8
Asv15;Bacteroides	0	0	1	0	prev0.8
Asv152;Oscillibacter	0	0	0	1	prev0.8
Asv158;Intestinibacter	0	0	1	1	prev0.8
Asv16;Haemophilus	0	1	1	1	prev0.8
Asv166;Clostridium sensu stricto 1	0	0	1	1	prev0.8
Asv167;Romboutsia	0	0	1	1	prev0.8
Asv17;Prevotella_7	0	1	0	0	prev0.8
Asv172;Anaerostipes	0	0	0	1	prev0.8
Asv18;Dialister	0	0	1	0	prev0.8
Asv180;Porphyromonas	0	1	0	0	prev0.8
Asv19;Gemella	1	1	0	0	prev0.8
Asv2;Faecalibacterium	0	0	1	1	prev0.8
Asv20;Escherichia-Shigella	0	0	1	1	prev0.8
Asv209;Actinomyces	0	1	0	0	prev0.8
Asv215;Erysipelatoclostridium	0	0	1	1	prev0.8
Asv22;Bifidobacterium	0	0	1	0	prev0.8
Asv23;Roseburia	0	0	1	1	prev0.8
Asv234;Ruminococcaceae_u	0	0	1	1	prev0.8
Asv24;Alistipes	0	0	0	1	prev0.8
Asv25;Streptococcus	1	1	1	1	prev0.8
Asv250;Hungatella	0	0	1	1	prev0.8
Asv252;Lautropia	1	0	0	0	prev0.8
Asv257;[Clostridium] innocuum group	0	0	1	1	prev0.8
Asv267;[Eubacterium] hallii group	0	0	0	1	prev0.8
Asv268;UBA1819	0	0	0	1	prev0.8
Asv27;Bifidobacterium	0	0	1	1	prev0.8
Asv274;Rothia	1	0	0	0	prev0.8
Asv28;Faecalibacterium	0	0	1	0	prev0.8
Asv287;Eggerthella	0	0	1	1	prev0.8
Asv29;Alloprevotella	1	1	0	0	prev0.8
Asv292;Lachnoclostridium	0	0	0	1	prev0.8
Asv3;Bacteroides	0	0	0	1	prev0.8
Asv311;Lachnospiraceae NK4A136 group	0	0	1	1	prev0.8
Asv33;Agathobacter	0	0	1	1	prev0.8
Asv37;Neisseria	0	1	0	0	prev0.8
Asv39;Blautia	0	0	1	1	prev0.8
Asv4;Streptococcus	1	1	1	0	prev0.8
Asv41;Bacteroides	0	0	1	0	prev0.8
Asv42;Ruminococcus	0	0	1	1	prev0.8
Asv45;Ruminococcus	0	0	0	1	prev0.8
Asv47;[Ruminococcus] gnavus group	0	0	1	1	prev0.8
Asv49;Agathobacter	0	0	1	1	prev0.8
Asv5;Faecalibacterium	0	0	1	1	prev0.8
Asv506;Anaerotruncus	0	0	1	0	prev0.8
Asv51;Granulicatella	1	1	0	0	prev0.8
Asv53;Actinomyces	1	0	0	0	prev0.8
Asv61;Granulicatella	1	1	0	0	prev0.8
Asv62;Actinomyces	0	1	0	0	prev0.8
Asv64;Streptococcus	1	1	1	0	prev0.8
Asv657;Terrisporobacter	0	0	1	0	prev0.8
Asv67;Blautia	0	0	0	1	prev0.8
Asv71;Blautia	0	0	0	1	prev0.8
Asv77;Flavonifractor	0	0	1	1	prev0.8
Asv8;Haemophilus	1	1	0	0	prev0.8
Asv85;Veillonella	0	1	0	0	prev0.8
Asv86;Lachnospiraceae NK4A136 group	0	0	1	1	prev0.8
Asv88;Fusicatenibacter	0	0	1	1	prev0.8
Asv89;Veillonella	0	0	0	1	prev0.8
Asv95;Lachnospiraceae UCG-004	0	0	0	1	prev0.8

Supplementary Table S3. Results of ANCOM-BC2 analysis for the top 20 genera in nasal and stool samples of healthy controls and wheezers showing effect sizes (log-fold change; LFC), 95% confidence intervals, and p values.

Taxon	LFC	CI_low	CI_high	p_value	p_adjusted	material	comparison
Alloprevotella	-0.34	-3.41	2.74	0.83	0.99	nose	healthy vs wheeze
Bacteroides	0.02	-1.38	1.42	0.98	0.99	nose	healthy vs wheeze
Fusobacterium	0.38	-2.73	3.48	0.81	0.99	nose	healthy vs wheeze
Gemella	0.66	-2.15	3.47	0.65	0.99	nose	healthy vs wheeze
Haemophilus	-1.40	-3.35	0.55	0.16	0.99	nose	healthy vs wheeze
Moraxella	-0.96	-5.10	3.18	0.65	0.99	nose	healthy vs wheeze
Neisseria	0.06	-2.90	3.02	0.97	0.99	nose	healthy vs wheeze
Prevotella_7	-0.39	-3.60	2.83	0.81	0.99	nose	healthy vs wheeze
Rothia	-0.41	-3.24	2.43	0.78	0.99	nose	healthy vs wheeze
Streptococcus	-0.41	-2.19	1.37	0.65	0.99	nose	healthy vs wheeze
Veillonella	0.26	-2.92	3.44	0.87	0.99	nose	healthy vs wheeze
Agathobacter	-0.32	-2.72	2.08	0.80	0.99	stool	healthy vs wheeze
Akkermansia	0.52	-2.51	3.55	0.74	0.99	stool	healthy vs wheeze
Alistipes	1.77	-0.32	3.86	0.10	0.99	stool	healthy vs wheeze
Bacteroides	-0.27	-1.05	0.52	0.50	0.99	stool	healthy vs wheeze
Bifidobacterium	0.52	-0.60	1.63	0.36	0.99	stool	healthy vs wheeze
Blautia	0.14	-0.80	1.08	0.77	0.99	stool	healthy vs wheeze
Faecalibacterium	0.65	-0.63	1.93	0.32	0.99	stool	healthy vs wheeze
Fusobacterium	0.27	-1.97	2.51	0.81	0.99	stool	healthy vs wheeze
Gemella	0.10	-0.77	0.96	0.83	0.99	stool	healthy vs wheeze
Haemophilus	-0.71	-2.14	0.72	0.33	0.99	stool	healthy vs wheeze
Neisseria	-0.02	-0.86	0.82	0.96	0.99	stool	healthy vs wheeze
Roseburia	0.02	-2.30	2.34	0.99	0.99	stool	healthy vs wheeze
Rothia	0.09	-1.14	1.33	0.88	0.99	stool	healthy vs wheeze
Ruminococcus	-0.51	-2.27	1.25	0.57	0.99	stool	healthy vs wheeze
Streptococcus	-0.07	-1.30	1.17	0.91	0.99	stool	healthy vs wheeze
Subdoligranulum	-1.56	-3.54	0.42	0.12	0.99	stool	healthy vs wheeze
Veillonella	0.22	-1.42	1.85	0.80	0.99	stool	healthy vs wheeze

Supplementary Table S4a. Results of Spearman correlations between beta-dispersion and bacterial genera (20 strongest correlations) in nasal samples of healthy controls and wheezers showing effect sizes (Spearman rho), 95% confidence intervals, and p values.

Taxon	Rho	CI_low	CI_high	p_value	p_adjusted	material	group
Actinomyces	-0.214	-0.842	0.615	0.610	0.809	nose	healthy
Alloprevotella	-0.405	-0.923	0.338	0.320	0.798	nose	healthy
Bergeyella	-0.476	-1.000	0.350	0.233	0.798	nose	healthy
Campylobacter	-0.439	-0.893	0.242	0.276	0.798	nose	healthy
Capnocytophaga	-0.790	-1.000	-0.095	0.020	0.798	nose	healthy
Catonella	-0.586	-1.000	0.125	0.127	0.798	nose	healthy
Fusobacterium	-0.381	-0.924	0.385	0.352	0.802	nose	healthy
Gemella	-0.214	-1.000	0.625	0.610	0.809	nose	healthy
Granulicatella	-0.690	-1.000	0.097	0.058	0.798	nose	healthy
Haemophilus	0.238	-0.722	0.899	0.570	0.809	nose	healthy
Lachnoanaerobaculum	-0.561	-0.942	0.261	0.148	0.798	nose	healthy
Lautropia	-0.786	-1.000	-0.174	0.021	0.798	nose	healthy
Leptotrichia	-0.238	-0.895	0.603	0.570	0.809	nose	healthy
Moraxella	0.143	-0.592	0.817	0.736	0.900	nose	healthy
Neisseria	-0.407	-0.975	0.600	0.317	0.798	nose	healthy
Porphyromonas	-0.381	-0.924	0.416	0.352	0.802	nose	healthy
Prevotella	-0.476	-1.000	0.342	0.233	0.798	nose	healthy
Prevotella_7	-0.287	-0.924	0.491	0.490	0.809	nose	healthy
Stomatobaculum	-0.512	-0.937	0.215	0.194	0.798	nose	healthy
Veillonella	-0.095	-0.750	0.787	0.823	0.900	nose	healthy
Actinomyces	-0.703	-0.895	-0.246	0.002	0.029	nose	wheeze
Alloprevotella	-0.660	-0.870	-0.180	0.004	0.047	nose	wheeze
Bergeyella	-0.662	-0.936	-0.215	0.004	0.047	nose	wheeze
Campylobacter	-0.716	-0.873	-0.225	0.001	0.026	nose	wheeze
Capnocytophaga	-0.700	-0.906	-0.245	0.002	0.029	nose	wheeze
Catonella	-0.687	-0.885	-0.278	0.002	0.034	nose	wheeze
Fusobacterium	-0.747	-0.931	-0.332	<0.001	0.015	nose	wheeze
Gemella	-0.765	-0.955	-0.311	<0.001	0.011	nose	wheeze
Granulicatella	-0.748	-0.953	-0.404	<0.001	0.015	nose	wheeze
Haemophilus	-0.787	-0.942	-0.501	<0.001	0.007	nose	wheeze
Lachnoanaerobaculum	-0.834	-0.944	-0.505	<0.001	0.002	nose	wheeze
Lautropia	-0.850	-0.940	-0.635	<0.001	0.001	nose	wheeze
Leptotrichia	-0.808	-0.952	-0.390	<0.001	0.004	nose	wheeze
Moraxella	0.640	0.068	0.919	0.006	0.064	nose	wheeze
Neisseria	-0.691	-0.938	-0.207	0.002	0.033	nose	wheeze
Porphyromonas	-0.907	-0.993	-0.658	0.000	<0.001	nose	wheeze
Prevotella	-0.821	-0.948	-0.438	<0.001	0.003	nose	wheeze
Prevotella_7	-0.674	-0.868	-0.183	0.003	0.041	nose	wheeze
Stomatobaculum	-0.584	-0.838	-0.132	0.014	0.120	nose	wheeze
Veillonella	-0.743	-0.895	-0.339	<0.001	0.015	nose	wheeze

Supplementary Table S4b. Results of Spearman correlations between beta-dispersion and bacterial genera (20 strongest correlations) in stool samples of healthy controls and wheezers showing effect sizes (Spearman rho), 95% confidence intervals, and p values.

Taxon	Rho	CI_low	CI_high	p_value	p_adjusted	material	group
Actinomyces	0.647	-0.148	0.975	0.083	0.762	stool	healthy
Bacteroides	-0.071	-0.747	0.755	0.867	0.971	stool	healthy
CAG.56	0.082	-0.519	0.598	0.846	0.971	stool	healthy
Citrobacter	0.577	0.581	0.877	0.134	0.762	stool	healthy
Clostridium.methylpentosum.group_u	-0.592	-0.877	-0.174	0.122	0.762	stool	healthy
Coprobacter	-0.078	-0.809	0.773	0.854	0.971	stool	healthy
Eubacterium.eligens.group	-0.623	-0.988	0.313	0.099	0.762	stool	healthy
Eubacterium.fissicatena.group	0.343	-0.282	0.793	0.406	0.971	stool	healthy
Eubacterium.hallii.group	-0.359	-0.960	0.519	0.382	0.971	stool	healthy
Faecalibacterium	-0.619	-1.000	0.156	0.102	0.762	stool	healthy
Lachnospiraceae.FCS020.group	0.265	-0.347	0.934	0.526	0.971	stool	healthy
Lachnospiraceae.UCG.004	-0.381	-1.000	0.763	0.352	0.971	stool	healthy
Prevotella_7	0.082	-0.463	0.595	0.846	0.971	stool	healthy
Robinsoniella	-0.082	-0.547	0.442	0.846	0.971	stool	healthy
Romboutsia	0.048	-1.000	0.889	0.911	0.971	stool	healthy
Roseburia	-0.333	-1.000	0.600	0.420	0.971	stool	healthy
Sellimonas	-0.683	-1.000	0.126	0.062	0.762	stool	healthy
UC5.1.2E3	-0.247	-0.778	0.257	0.555	0.971	stool	healthy
Actinomyces	0.424	-0.130	0.752	0.090	0.674	stool	wheeze
Atopobium	0.435	0.134	0.746	0.081	0.674	stool	wheeze
Bacteroides	-0.630	-0.860	-0.178	0.007	0.493	stool	wheeze
CAG.56	0.484	0.047	0.822	0.049	0.674	stool	wheeze
Citrobacter	0.380	0.040	0.699	0.133	0.698	stool	wheeze
Clostridium.methylpentosum.group_u	-0.441	-0.707	-0.033	0.076	0.674	stool	wheeze
Coprobacter	-0.420	-0.700	-0.051	0.093	0.674	stool	wheeze
Eubacterium.eligens.group	-0.541	-0.870	-0.032	0.025	0.674	stool	wheeze
Eubacterium.fissicatena.group	0.301	-0.210	0.815	0.241	0.716	stool	wheeze
Eubacterium.hallii.group	0.640	0.240	0.910	0.006	0.493	stool	wheeze
Faecalibacterium	-0.654	-0.890	-0.153	0.004	0.493	stool	wheeze
Lachnospiraceae.FCS020.group	0.409	-0.063	0.768	0.103	0.674	stool	wheeze
Lachnospiraceae.UCG.004	-0.407	-0.744	0.079	0.105	0.674	stool	wheeze
Marvinbryantia	0.486	0.307	0.748	0.048	0.674	stool	wheeze
Prevotella_7	0.474	0.185	0.752	0.055	0.674	stool	wheeze
Robinsoniella	0.609	0.226	0.774	0.009	0.519	stool	wheeze
Romboutsia	0.528	0.025	0.805	0.029	0.674	stool	wheeze
Roseburia	-0.346	-0.696	0.148	0.174	0.699	stool	wheeze
Sellimonas	-0.284	-0.717	0.258	0.269	0.777	stool	wheeze
UC5.1.2E3	-0.398	-0.666	-0.103	0.113	0.674	stool	wheeze

Supplementary Table S5. Summary of baseline characteristics of wheezing children further stratified by microbial community composition distances.

		nose			stool		
Characteristic	Overall N = 17	healthy-like N = 9	divergent N = 8	p value [1]	healthy-like N = 10	divergent N = 7	p value [1]
Age (year)	3.00 (1.90-3.40)	3.00 (2.60-3.30)	2.55 (1.50-3.70)	0.772	3.00 (1.90-3.40)	3.20 (1.50-3.70)	0.807
Female	4 (24%)	4 (44%)	0 (0%)	0.082	3 (30%)	1 (14%)	0.603
BMI (kg/m^3)	16.98 (15.50-18.20)	17.40 (15.50-18.20)	16.67 (15.76-18.08)	0.501	16.84 (15.30-18.20)	17.90 (15.50-18.26)	0.696
Any sibling	12 (71%)	7 (78%)	5 (63%)	0.620	7 (70%)	5 (71%)	>0.999
Daycare	15 (88%)	7 (78%)	8 (100%)	0.471	9 (90%)	6 (86%)	>0.999
Pet in household (cat/dog)	7 (41%)	4 (44%)	3 (38%)	>0.999	2 (20%)	5 (71%)	0.058
Asthma or recurrent spastic/obstructive bronchitis - DD	2 (13%)	2 (22%)	0 (0%)	0.475	0 (0%)	2 (33%)	0.125
Missing	1	0	1		0	1	
Hayfever - DD	1 (5.9%)	1 (11%)	0 (0%)	>0.999	1 (10%)	0 (0%)	>0.999
Eczema - DD	2 (13%)	1 (11%)	1 (14%)	>0.999	1 (11%)	1 (14%)	>0.999
Missing	1	0	1		1	0	
Food allergy - DD	0 (0%)	0 (0%)	0 (0%)		0 (0%)	0 (0%)	-- [2]
Birthmode				0.091			0.449
Vaginal delivery	8 (47%)	5 (56%)	3 (38%)		6 (60%)	2 (29%)	
Primary cesarean section	6 (35%)	1 (11%)	5 (63%)		2 (20%)	4 (57%)	
Secondary cesarean section	1 (5.9%)	1 (11%)	0 (0%)		1 (10%)	0 (0%)	
Operative vaginal delivery	2 (12%)	2 (22%)	0 (0%)		1 (10%)	1 (14%)	
Any exclusive breastfeeding ever	14 (82%)	7 (78%)	7 (88%)	>0.999	8 (80%)	6 (86%)	>0.999
Any hypoallergenic formula ever	8 (47%)	5 (56%)	3 (38%)	0.637	5 (50%)	3 (43%)	>0.999
Prior RSV infection ever	7 (47%)	3 (38%)	4 (57%)	0.619	3 (38%)	4 (57%)	0.619
Missing	2	1	1		2	0	
Antibiotics given for recurrent cough ever	1 (33%)	1 (50%)	0 (0%)	>0.999	1 (33%)	NA	-- [2]
Missing	14	7	7		7	7	
Systemic steroids ever	9 (53%)	4 (44%)	5 (63%)	0.637	4 (40%)	5 (71%)	0.335
ICS ever	13 (76%)	6 (67%)	7 (88%)	0.576	8 (80%)	5 (71%)	>0.999
Hb (g/dl)	11.90 (11.45-12.25)	11.90 (11.50-12.25)	11.75 (11.45-12.40)	0.958	11.90 (11.50-12.30)	11.90 (11.30-12.20)	0.558
Missing	1	1	0		1	0	
Thrombocytes (10^3/µl)	403 (372-443)	403 (374-430)	418 (318-449)	0.958	397 (370-443)	408 (374-455)	0.491
Missing	1	1	0		1	0	
Leucocytes (10^3/µl)	9.27 (8.24-11.65)	10.80 (9.27-11.95)	8.52 (7.94-9.91)	0.074	9.40 (8.25-10.78)	9.03 (8.23-12.20)	0.874
Missing	1	1	0		1	0	
Neutrophils (%)	42 (36-55)	48 (36-55)	39 (33-51)	0.563	40 (37-47)	55 (21-60)	0.427
Missing	1	1	0		1	0	
Lymphocytes (%)	40 (36-47)	38 (33-42)	47 (38-59)	0.115	41 (39-48)	38 (30-45)	0.427
Missing	1	1	0		1	0	
Eosinophils (%)	1.50 (0.78-2.40)	1.14 (0.47-2.20)	2.00 (1.00-3.90)	0.295	2.00 (1.00-2.40)	0.90 (0.50-2.00)	0.375
Missing	2	1	1		1	1	
Basophils (%)	0.55 (0.08-1.00)	0.25 (0.04-0.75)	0.85 (0.50-1.00)	0.194	0.70 (0.08-1.00)	0.50 (0.10-0.60)	0.841
Missing	3	1	2		1	2	
IgE (IU/ml)	19 (9-82)	19 (9-93)	26 (9-43)	0.724	19 (9-43)	37 (6-82)	0.724
Missing	2	0	2		1	1	
Atopy (spec. IgE to any alleren ≥0.7 kU/L)	5 (29%)	3 (33%)	2 (25%)	>0.999	4 (40%)	1 (14%)	0.338

Values are summarized as median and interquartile range (Q1-Q3) in brackets, or as total numbers per condition level and percentage in brackets.
DD = parental report of doctor's diagnosis
[1]Kruskal-Wallis rank sum test; Fisher's exact test
[2]p value not calculated due to insufficient variability (single-level data) and/or missing values

Supplementary Table S6. Results of alpha diversity analysis and beta-dispersion analysis of wheezing children further stratified by microbial community composition distances. Shown are effect sizes (beta), 95% confidence intervals, and p values.

Measure	Material	Beta	CI_low	CI_high	p.value	p_adjusted	comparison	group
Observed	stool	-0.008	0.923	1.067	0.835	0.835	healthy-like vs divergent	wheeze
Observed	nose	-0.290	0.670	0.836	<0.001	<0.001	healthy-like vs divergent	wheeze
Shannon	stool	0.109	-0.106	0.323	0.320	0.384	healthy-like vs divergent	wheeze
Shannon	nose	-2.587	-3.195	-1.979	<0.001	<0.001	healthy-like vs divergent	wheeze
Evenness	stool	0.094	-0.062	0.251	0.237	0.355	healthy-like vs divergent	wheeze
Evenness	nose	-2.413	-3.079	-1.747	<0.001	<0.001	healthy-like vs divergent	wheeze
Betadispersion	stool	0.492	0.246	0.738	<0.001	<0.001	healthy-like vs divergent	wheeze
Betadispersion	nose	0.626	0.361	0.891	<0.001	<0.001	healthy-like vs divergent	wheeze

Supplementary Table S7. Results of ANCOM-BC2 analysis for the top 20 genera in nasal and stool samples of wheezing children further stratified by microbial community composition distances. Shown are effect sizes (log-fold change; LFC), 95% confidence intervals, and p values.

Taxon	LFC	CI_low	CI_high	p_value	p_adjusted	material	comparison	group
Alloprevotella	-3.728	-4.775	-2.681	<0.001	<0.001	nose	healthy-like vs divergent	wheeze
Bacteroides	2.910	2.298	3.522	0.001	0.002	nose	healthy-like vs divergent	wheeze
Fusobacterium	-3.052	-4.289	-1.815	0.001	0.001	nose	healthy-like vs divergent	wheeze
Gemella	-1.907	-3.444	-0.370	0.030	0.038	nose	healthy-like vs divergent	wheeze
Granulicatella	-1.779	-3.045	-0.513	0.016	0.021	nose	healthy-like vs divergent	wheeze
Haemophilus	-0.577	-2.292	1.138	0.521	0.535	nose	healthy-like vs divergent	wheeze
Moraxella	9.038	7.123	10.953	<0.001	<0.001	nose	healthy-like vs divergent	wheeze
Neisseria	-2.080	-2.915	-1.245	<0.001	0.001	nose	healthy-like vs divergent	wheeze
Prevotella_7	-3.991	-4.769	-3.213	<0.001	<0.001	nose	healthy-like vs divergent	wheeze
Rothia	-1.347	-2.617	-0.077	0.062	0.073	nose	healthy-like vs divergent	wheeze
Streptococcus	-0.406	-1.478	0.666	0.471	0.497	nose	healthy-like vs divergent	wheeze
Veillonella	-3.154	-4.103	-2.205	<0.001	<0.001	nose	healthy-like vs divergent	wheeze
Akkermansia	0.580	-2.319	3.479	0.701	0.915	stool	healthy-like vs divergent	wheeze
Alistipes	-1.733	-3.140	-0.326	0.031	0.255	stool	healthy-like vs divergent	wheeze
Bacteroides	-0.561	-1.245	0.123	0.132	0.414	stool	healthy-like vs divergent	wheeze
Bifidobacterium	0.471	-0.772	1.714	0.471	0.822	stool	healthy-like vs divergent	wheeze
Blautia	0.096	-0.788	0.980	0.834	0.961	stool	healthy-like vs divergent	wheeze
Faecalibacterium	-0.626	-1.302	0.050	0.093	0.342	stool	healthy-like vs divergent	wheeze
Fusobacterium	2.225	0.635	3.815	0.029	0.255	stool	healthy-like vs divergent	wheeze
Gemella	0.471	-0.176	1.118	0.214	0.525	stool	healthy-like vs divergent	wheeze
Haemophilus	-0.245	-1.709	1.219	0.748	0.924	stool	healthy-like vs divergent	wheeze
Neisseria	0.288	-0.559	1.135	0.541	0.860	stool	healthy-like vs divergent	wheeze
Roseburia	0.426	-1.062	1.914	0.584	0.865	stool	healthy-like vs divergent	wheeze
Ruminococcus	-1.530	-3.400	0.340	0.133	0.414	stool	healthy-like vs divergent	wheeze
Streptococcus	-0.548	-1.749	0.653	0.388	0.716	stool	healthy-like vs divergent	wheeze
Subdoligranulum	0.248	-1.126	1.622	0.730	0.922	stool	healthy-like vs divergent	wheeze
Veillonella	-0.499	-1.985	0.987	0.522	0.850	stool	healthy-like vs divergent	wheeze

Supplementary Table S8. Results of ANCOM-BC2 analysis for the top 20 ASV within nasal and stool samples of wheezing children further stratified by microbial community composition distances. Shown are effect sizes (log-fold change; LFC), 95% confidence intervals, and p values.

Taxon	LFC	CI_low	CI_high	p_value	p_adjusted	material	comparison	group
Asv1;Moraxella	8.090	5.499	10.681	<0.001	0.003	nose	healthy-like vs divergent	wheeze
Asv97;Neisseriaceae_u	3.393	2.509	4.277	0.005	0.016	nose	healthy-like vs divergent	wheeze
Asv582;Archaea_u	2.957	1.769	4.145	0.003	0.012	nose	healthy-like vs divergent	wheeze
Asv51;Granulicatella	-1.677	-2.943	-0.411	0.025	0.049	nose	healthy-like vs divergent	wheeze
Asv114;Porphyromonas	-1.907	-3.275	-0.539	0.021	0.045	nose	healthy-like vs divergent	wheeze
Asv64;Streptococcus	-1.974	-3.370	-0.578	0.020	0.045	nose	healthy-like vs divergent	wheeze
Asv85;Veillonella	-1.989	-3.424	-0.554	0.022	0.045	nose	healthy-like vs divergent	wheeze
Asv4;Streptococcus	-2.184	-3.634	-0.734	0.011	0.033	nose	healthy-like vs divergent	wheeze
Asv124;Porphyromonas	-2.221	-3.597	-0.845	0.019	0.045	nose	healthy-like vs divergent	wheeze
Asv305;Actinomyces	-2.424	-3.408	-1.440	0.001	0.007	nose	healthy-like vs divergent	wheeze
Asv244;Capnocytophaga	-2.442	-4.114	-0.770	0.021	0.045	nose	healthy-like vs divergent	wheeze
Asv62;Actinomyces	-2.558	-3.901	-1.215	0.004	0.014	nose	healthy-like vs divergent	wheeze
Asv19;Gemella	-2.589	-4.431	-0.747	0.016	0.041	nose	healthy-like vs divergent	wheeze
Asv37;Neisseria	-2.994	-4.431	-1.557	0.002	0.010	nose	healthy-like vs divergent	wheeze
Asv53;Actinomyces	-3.165	-4.343	-1.987	0.001	0.005	nose	healthy-like vs divergent	wheeze
Asv162;Prevotella_7	-3.849	-4.951	-2.747	<0.001	0.003	nose	healthy-like vs divergent	wheeze
Asv10;Veillonella	-4.065	-5.684	-2.446	<0.001	0.004	nose	healthy-like vs divergent	wheeze
Asv29;Alloprevotella	-4.138	-5.428	-2.848	<0.001	0.002	nose	healthy-like vs divergent	wheeze
Asv32;Fusobacterium	-4.211	-5.591	-2.831	<0.001	0.003	nose	healthy-like vs divergent	wheeze
Asv17;Prevotella_7	-4.401	-5.644	-3.158	<0.001	0.002	nose	healthy-like vs divergent	wheeze
Asv140;Faecalibacterium	3.871	2.266	5.476	0.042	0.303	stool	healthy-like vs divergent	wheeze
Asv54;Bifidobacterium	2.858	1.894	3.822	0.001	0.094	stool	healthy-like vs divergent	wheeze
Asv194;Lachnoclostridium	2.749	1.642	3.856	0.008	0.160	stool	healthy-like vs divergent	wheeze
Asv538;Enterococcus	2.542	1.409	3.675	0.012	0.179	stool	healthy-like vs divergent	wheeze
Asv148;NK4A214 group	2.399	1.100	3.698	0.036	0.288	stool	healthy-like vs divergent	wheeze
Asv223;Intestinimonas	2.245	1.093	3.397	0.009	0.160	stool	healthy-like vs divergent	wheeze
Asv5;Faecalibacterium	2.045	0.259	3.831	0.043	0.303	stool	healthy-like vs divergent	wheeze
Asv601;Coprobacillus	1.791	0.868	2.714	0.009	0.160	stool	healthy-like vs divergent	wheeze
Asv167;Romboutsia	1.659	0.426	2.892	0.025	0.288	stool	healthy-like vs divergent	wheeze
Asv41;Bacteroides	1.616	0.577	2.655	0.028	0.288	stool	healthy-like vs divergent	wheeze
Asv150;Lachnospiraceae NK4A136 group	1.289	0.360	2.218	0.035	0.288	stool	healthy-like vs divergent	wheeze
Asv292;Lachnoclostridium	1.263	0.312	2.214	0.026	0.288	stool	healthy-like vs divergent	wheeze
Asv346;[Eubacterium] hallii group	1.217	0.451	1.983	0.012	0.179	stool	healthy-like vs divergent	wheeze
Asv141;Alistipes	-1.220	-1.855	-0.585	0.033	0.288	stool	healthy-like vs divergent	wheeze
Asv63;[Eubacterium] eligens group	-1.583	-2.814	-0.352	0.033	0.288	stool	healthy-like vs divergent	wheeze
Asv101;Clostridium sensu stricto 1	-1.677	-2.561	-0.793	0.007	0.160	stool	healthy-like vs divergent	wheeze
Asv445;Haemophilus	-1.866	-2.656	-1.076	0.006	0.160	stool	healthy-like vs divergent	wheeze
Asv100;[Ruminococcus] torques group	-2.554	-4.018	-1.090	0.008	0.160	stool	healthy-like vs divergent	wheeze
Asv2;Faecalibacterium	-2.720	-4.835	-0.605	0.027	0.288	stool	healthy-like vs divergent	wheeze
Asv145;[Eubacterium] coprostanoligenes group_u	-3.936	-5.110	-2.762	0.007	0.160	stool	healthy-like vs divergent	wheeze
